# FOXA2-initiated transcriptional activation of INHBA induced by methylmalonic acid promotes pancreatic neuroendocrine neoplasm progression

**DOI:** 10.1007/s00018-023-05084-0

**Published:** 2024-01-22

**Authors:** Chunhua Hu, Mujie Ye, Jianan Bai, Pengfei Liu, Feiyu Lu, Jinhao Chen, Yanling Xu, Lijun Yan, Ping Yu, Zequan Xiao, Danyang Gu, Lin Xu, Ye Tian, Qiyun Tang

**Affiliations:** 1 Department of Geriatric Gastroenterology, Neuroendocrine Tumor Center, Jiangsu Province Hospital, The First Affiliated Hospital of Nanjing Medical University, Institute of Neuroendocrine Tumor, Nanjing Medical University, No. 300 Guangzhou Road, Nanjing, China; 2 Department of Gastroenterology, Jiangyin People’s Hospital, Jiangyin, Jiangsu Province China; 3 Department of Gastroenterology, The Friendship Hospital of Ili Kazakh Autonomous Prefecture, Ili State, China

**Keywords:** Metabolic alterations, Tumor progression, Pancreatic neuroendocrine neoplasm, FOXA2, INHBA, Epithelial–mesenchymal transition

## Abstract

Pancreatic neuroendocrine neoplasms (PanNENs) are a group of highly heterogeneous neoplasms originating from the endocrine islet cells of the pancreas with characteristic neuroendocrine differentiation, more than 60% of which represent metastases when diagnosis, causing major tumor-related death. Metabolic alterations have been recognized as one of the hallmarks of tumor metastasis, providing attractive therapeutic targets. However, little is known about the molecular mechanism of metabolic changes regulating PanNEN progression. In this study, we first identified methylmalonic acid (MMA) as an oncometabolite for PanNEN progression, based on serum metabolomics of metastatic PanNEN compared with non-metastatic PanNEN patients. One of the key findings was the potentially novel mechanism of epithelial–mesenchymal transition (EMT) triggered by MMA. Inhibin βA (INHBA) was characterized as a key regulator of MMA-induced PanNEN progression according to transcriptomic analysis, which has been validated in vitro and in vivo. Mechanistically, INHBA was activated by FOXA2, a neuroendocrine (NE) specific transcription factor, which was initiated during MMA-induced progression. In addition, MMA-induced INHBA upregulation activated downstream MITF to regulate EMT-related genes in PanNEN cells. Collectively, these data suggest that activation of INHBA via FOXA2 promotes MITF-mediated EMT during MMA inducing PanNEN progression, which puts forward a novel therapeutic target for PanNENs.

**Graphical abstract:**

Methylmalonic acid (MMA), a serum oncometabolite, increased the expression of inhibin βA (INHBA) by the neuroendocrine-specific transcription factor, FOXA2 to induce MITF-mediated EMT during the progression of pancreatic neuroendocrine neoplasms (PanNENs), providing an actionable therapeutic vulnerability to metabolic therapy in PanNENs.

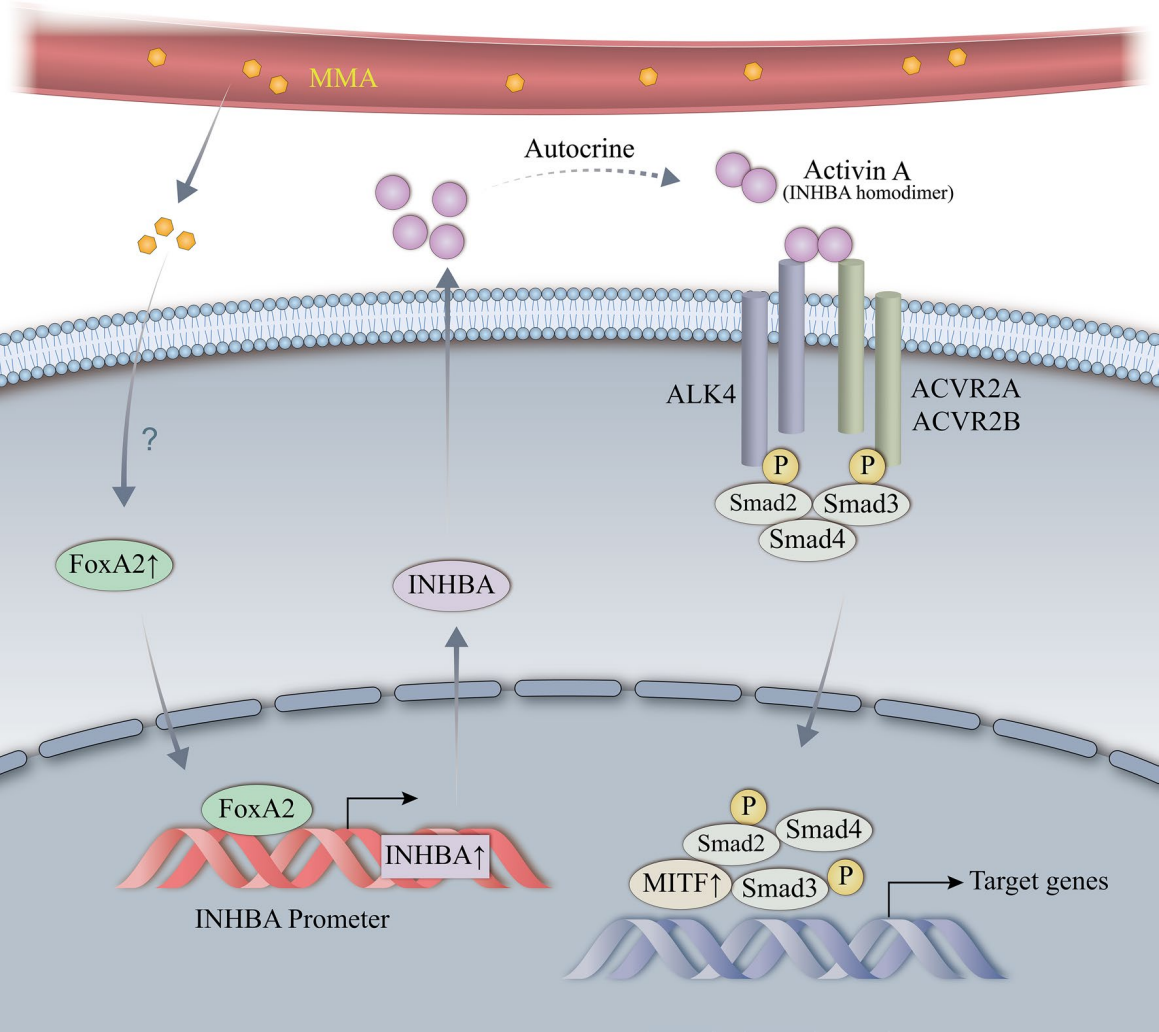

**Supplementary Information:**

The online version contains supplementary material available at 10.1007/s00018-023-05084-0.

## Introduction

Pancreatic neuroendocrine neoplasms (PanNENs) are a group of highly heterogeneous neoplasms originating from the endocrine islet cells of the pancreas with characteristic neuroendocrine differentiation [1]. Although PanNEN is the second most common type of pancreatic neoplasm, which accounts for 3–5% of all cases, the annual incidence is increasing more rapidly than any other histological type of pancreatic tumor [2]. While PanNEN was initially considered quite rare, the incidence has increased approximately fivefold over the past decades in the Surveillance, Epidemiology, and End Results (SEER) population-based database [3]. While PanNEN is often perceived as indolent, which is less aggressive than pancreatic ductal adenocarcinomas (PDAC), the prevalence of distant metastases at the time of initial diagnosis in PanNEN is surprisingly high (over 60%), and cancer-related death is generally caused by distant metastases [4, 5]. The liver is the most common site of metastasis with 50–80% of PanNENs having hepatic involvement, which might be explained by the classic “anatomical/ mechanical” hypothesis [6, 7]. The median overall survival (OS) of PanNEN patients in SEER is 235 months for patients with localized tumors and this decreased to 20 months in metastatic disease [8]. Therefore, there is an urgent need to better understand the biology of metastatic PanNEN and further explore the specific mechanism to identify novel molecularly targeted therapies.

The development of tumor metastasis is a complex process [9], and epithelial–mesenchymal transition (EMT) is the first critical step in the metastatic cascades [10], which is characterized by loss of epithelial markers E-cadherin, gaining mesenchymal markers such as vimentin and N-cadherin [11]. The understanding of the mechanism and prevention of the initiation of tumor metastasis is critical to identifying potential therapeutic targets [12]. Metabolic alterations have been recognized as one of the hallmarks of cancer cells [13], which provides a suitable environment for cancer cells to adapt and progress [14, 15]. Numerous studies have explored the association between altered tumor metabolism and the metastasis of various types of cancer [16–18]. There is a complex interaction between cellular metabolism and tumor metastasis, adapting to unique environment in the circulation and metastatic niche [19]. On the one hand, activation of oncogenic signaling pathways and oncogenic mutations could promote tumor progression by altering cell metabolism [20]. For example, EMT transcription factor Snail promoted malignancy of cancer cell by regulating cellular metabolic pathways [21]. On the other hand, changes in metabolism highly influence signaling networks and regulation of gene expression. Apart from nutrient consumption, metabolic changes could act as signaling molecules to directly regulate signaling pathways that drive tumor progression [22].

The accumulation of certain metabolites, known as oncometabolite, can drive tumor metastasis, in autocrine, paracrine, or endocrine fashions [23]. Metabolites produced by tumor cells can have a profound effect on metastatic cascade, including EMT and metastatic colonization at distant sites [24]. Several metabolites have been identified to regulate EMT facilitating tumor progression. Pyruvate can dictate the collagen modification in the extracellular matrix (ECM) of the lungs to promote metastasis [25]. 2-Hydroxyglutarate (2-HG), accumulating in tumors with isocitrate dehydrogenase 1 or 2 (IDH1/2) mutations, promoted ZEB1-mediated EMT [26]. The accumulation of α-Ketoglutarate led to EMT in cancer [27]. Moreover, Gomes et al. have previously demonstrated that the metabolite of aged serum was a predominant oncometabolite for cancer progression in breast and lung cancer [28].

The complex interaction between metabolic alterations and metastatic signalings provides a new perspective for targeting tumor metastasis. However, there is little research about the effects of metabolites on the progression of PanNEN. Thus, the aim of this study was to screen and identify the differential metabolites that correlate with the metastasis of PanNEN based on serum metabolomics profiling, and clarify the specific molecular mechanism.

## Materials and methods

### Human serum and neuroendocrine tumor specimens

Human serum from five PanNEN patients with metastasis (M) and five non-metastatic PanNEN (NM) were obtained from Jiangsu Province Hospital (Nanjing, China). A total of five PanNEN tissues and matching paratumoral tissues were obtained from individuals who underwent surgical resection at Jiangsu Province Hospital. The study was approved by the Jiangsu Province Hospital Research Ethics Committee (2023-SR-230) and all patients signed written informed consent prior to surgery. The serum VitB12 level of PanNEN patients was detected by ELISA kit (CUSABIO, CSB-E07903h) according to the manufacturer’s instructions.

### Targeted metabolomics and data analysis

Targeted gas chromatography–tandem mass spectrometry (GC–MS/MS) was performed using a Trace1310 gas chromatography system coupled to a TSQ9000 Mass spectrometer with an EI source (Thermo Fisher Scientific, USA). Data were acquired using selective reaction monitoring (SRM) mode by triple quadrupole mass spectrometry. Peak areas from the total ion current for each metabolite SRM transition were integrated using TraceFinder 4.1 General Quan software. Statistical analysis of the data was carried out using the R-software (v3.6.0). Targeted metabolomics was performed by Shanghai Luming Biotech Co. Ltd. The original data were normalized to the median of the entire metabolome in each sample and log transformed prior to further analysis (Supplementary Table 1).

### Cell culture and treatment

The QGP-1 cell line was purchased from the JCRB Cell Bank (JCRB0183), which was generated from a primary pancreatic neuroendocrine cancer. The BON-1 cell line was a generous gift from Professor Yu Xianjun, Affiliated Cancer Hospital of Fudan University. Human Pancreatic Nestin-Expressing ductal cells line (HPNE) was obtained from the ATCC (CBP60857). Murine neuroendocrine tumor cell line STC-1 was purchased from the ATCC (CRL-3254). Moreover, we constructed the primary human PanNEN cells (named IPC619) isolated from neoplasm tissues of a consenting female patient diagnosed with non-functional-PanNET, using the methods already described [29]. We performed STR testing and evaluated neuroendocrine associated markers in IPC619 cells by western blotting and cellular immunofluorescence assay (Fig. S1). QGP-1 and HPNE cell lines were cultured in RPMI-1640 (Gibco, USA), BON-1 cell lines were cultured in DMEM/F-12 (1:1) (Gibco, USA). IPC619 cells were maintained in McCoy’s 5A (Gibco), and STC-1 cells were maintained in Dulbecco’s Modified Eagle Medium (DMEM, Gibco), supplemented with 10% fetal bovine serum (FBS) and 1% penicillin/streptomycin at 37℃, 5% CO_2_.

To evaluate the effects of the significantly elevated metabolites in the serum of the metastatic PanNEN patients, cells were treated with 1 mM methylmalonic acid (MMA; Sigma-Aldrich), 1 mM 2-hydroxyhexanoic acid (MCE), 1 mM uracil (MCE) or vehicle. To determine the optimal conditions for MMA stimulation of PanNEN cells in the subsequent experiments, cells were treated with indicated concentrations of MMA for the time frames indicated. To test the effects of INHBA on EMT, cells were treated with indicated concentrations of recombinant human INHBA (rh-INHBA; ACRO Biosystems) for 24 h after serum starvation for 12 h. To inhibit activin A and TGF-β signaling, after serum starvation for 12 h, cells were treated with 10 μM SB431542 (Selleck Chem, USA) for 2 h, after which MMA was added to cells, along with reintroduction of FBS, and then maintained with the inhibitor for the duration of MMA treatment in the presence of normal serum. To test the effects of the top upregulated metabolites in the aged serum, cells were treated with 5 mM MMA (Sigma-Aldrich, USA), 5 mM quinolinate (QA, Sigma-Aldrich), 5 mM phosphoenolpyruvate (PEP, Sigma-Aldrich). For all acidic treatments, 25 mM HEPES (Livning, Beijing, China) was added to the treatment media to buffer potential changes in pH, and the media were replaced every day during the treatments.

### RNA sequencing

Total RNA was obtained from QGP-1 cells with TRIzol reagent (Life, USA). Illumina Paired End Sample Prep kits (Illumina, Inc.) were used to prepare an Illumina libraries. Each cDNA library was sequenced using an Illumina Hiseq 4000 (cat. no. PE150; Illumina, Inc.). Differential expression levels of mRNA between groups were evaluated and subsequent GO and KEGG analyses were performed.

### Animal experiments

Male BALB/c nude mice (4–6 weeks of age) were bred in laminar flow hoods using plastic cages with filter caps. Animal research procedures in vivo were performed in compliance with Nanjing Medical University Institutional Animal Care and Use Committee (IACUC) protocols (IACUC-2204053). PanNEN cells were pretreated with MMA or SB431542 combined with MMA for 10 days before injected subcutaneously or into the tail vein and then continued the treatment in vivo, as described before [28]. To achieve increased circulatory MMA concentrations and verify the effect of MMA on tumor progression in vivo, mice of MMA-induced group were treated with MMA (200 μg MMA/g/day) through the drinking water. SB431542 0.3 mg/mouse was intraperitoneally injected three times a week. The tumor xenograft model was constructed by subcutaneously injecting 5 × 10^6^ QGP-1 cells suspended in 100 μl PBS. The mice were sacrificed after 4 weeks, and tumor volumes were calculated using the formula: length × width^2^/2. For in vivo metastasis assay, the treated cells (1 × 10^5^ cells suspended in 100 μl PBS) were injected into tail veins for the establishment of the liver metastatic model. Mice were sacrificed 8 weeks post-injection and the liver metastatic foci were obtained for further analysis. The serum MMA level of mice was measured based on LC–MS.

### Chromatin immunoprecipitation (ChIP)-qPCR

ChIP assay was performed using the ChIP assay kit (Beyotime) according to the manufacturer’s instruction. The primers for ChIP-qPCR are listed in Supplementary Table 2.

### Dual luciferase reporter assay

293T cells were co-transfected with the luciferase reporter plasmids of INHBA-wt or INHBA-muts, pRL-TK plasmid (Genomeditech) and FOXA2 overexpression/ empty vector for 48 h. Then the cells were lysed, and luciferase activity was measured using the Dual-Luciferase Reporter Gene Assay Kit (Yeasen) according to the manufacturer’s instructions. The activity of firefly luciferase activity was normalized to the Renilla luciferase activity as control.

### Bioinformatic analysis

The potential transcript factor for INHBA was predicted based on RNA-seq data of MMA-induced QGP-1 cells via Metascape (https://metascape.org/). The possible targets of FOXA2 and SOX9 was predicted via hTFtarget (http://bioinfo.life.hust.edu.cn/hTFtarget/). The binding between FOXA2 and the INHBA promoter region was predicted via JASPAR (https://jaspar.genereg.net/). Expression profile data of INHBA analyed in this study were obtained from RNA-seq BAM file EGAS00001005024.

### Statistics analysis

The statistical analysis was performed using the GraphPad Prism7. Results were presented as mean ± SD. Pearson’s correlation analysis was used to analyze the correlation between two indices. The survival curves were plotted by Kaplan–Meier analysis and assessed by log-rank test. The two-tailed Student’s *t* test was used to determine significance, and *P* < 0.05 was considered statistically significant.

## Results

### Serum metabolomics analysis of PanNEN patients with or without metastasis

**Fig. 1:**
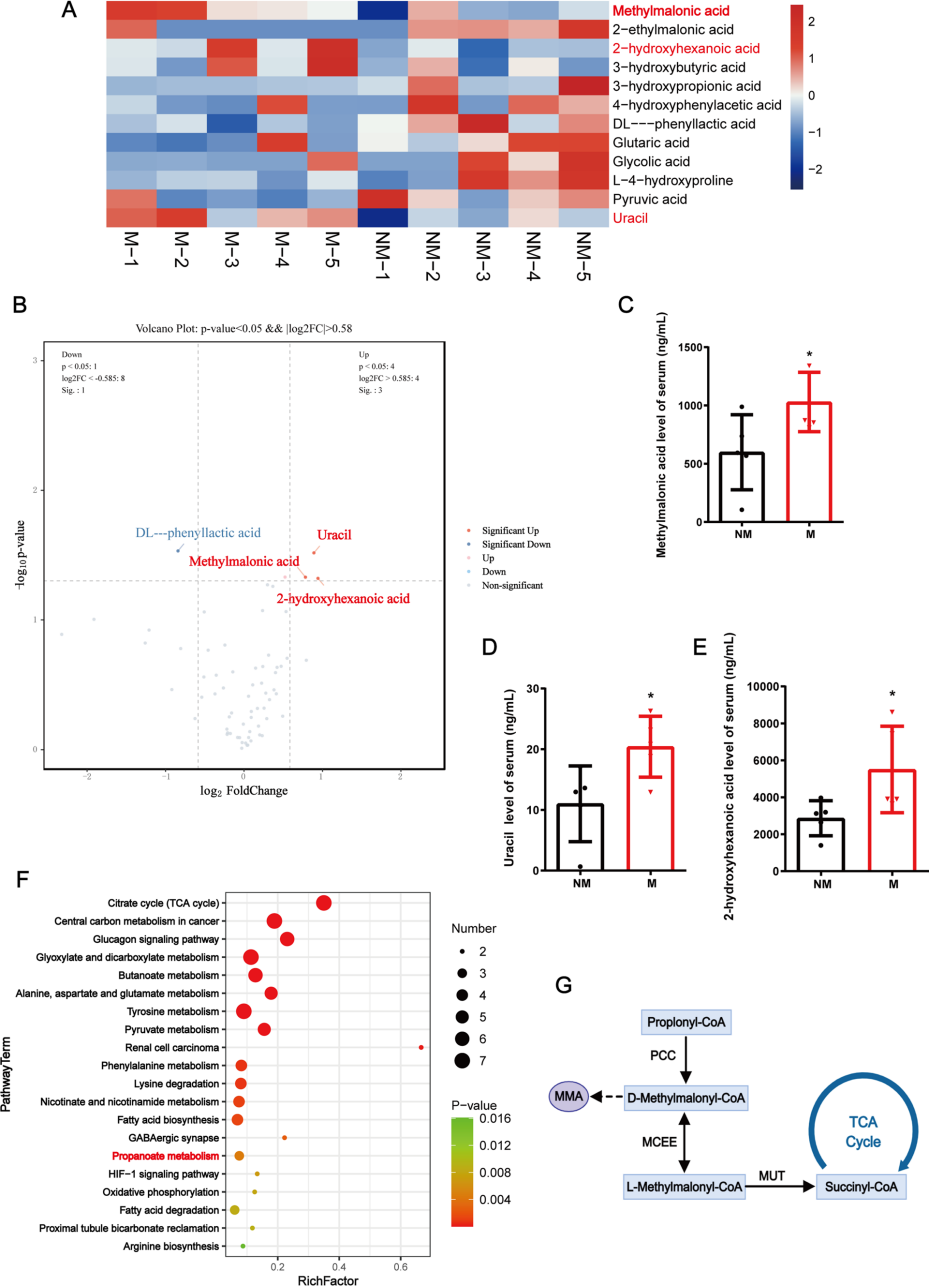
Identification of metabolites associated with PanNEN metastasis based on serum metabolomics analysis. **A** Heat map presents candidate metabolites with more than 1.5-fold change between PanNEN patients with metastasis (M) and non-metastatic PanNEN (NM), based on serum metabolomics analysis (*n* = 5 biologically independent samples). The red font represents significantly elevated metabolites. **B** Volcano plot summarizes the serum metabolomics analysis of PanNEN serum samples used in this study. **C–E** Comparison of serum metabolite concentrations in samples from metastatic (M) and non-metastatic PanNENs (NM). **F** Bubble map of metabolic pathway enrichment (Top 20). **G** Schematic representation of propanoate metabolism

To characterize the metabolic changes that promote tumor progression in patients with PanNEN, metabolic compositions of serum from PanNEN patients with or without metastasis were examined (Supplementary Table 1). Out of the 99 serum metabolites detected by targeted metabolomics, 38 metabolites were upregulated, of which only 3 metabolites were significantly elevated in the serum of patients with metastatic PanNENs: methylmalonic acid (MMA), uracil, and 2-hydroxyhexanoic acid (Fig. 1A, B). The serum concentrations of these three metabolites in metastatic PanNEN patients were approximately twice that of the non-metastatic group (Fig. 1C–E). Pathway enrichment analysis revealed that the propanoate metabolism pathway was enriched in the serum of patients with metastatic PanNEN (Fig. 1F). MMA is a by-product of propanoate metabolism pathway, which has been reported to promote metastasis in lung and breast cancers in previous studies (Fig. 1G).

It has been known that MMA levels can be significantly influenced by renal function and vitamin B12 (VitB12) deficiency. Therefore, we analyzed the renal function indicators of these patients and found no significant differences in estimated glomerular filtration rate (eGFR), blood urea nitrogen (BUN), and blood creatinine (Cr) levels between the two groups (Supplementary Table 1; Fig. S2A–C). Besides, we detected the serum VitB12 levels of these patients by ELISA and found that the serum VitB12 levels of metastatic PanNEN patients were significantly lower than that of patients with non-metastatic PanNEN (Supplementary Table 1; Fig. S2D), suggesting that serum MMA accumulation in patients with metastatic PanNEN might be related to VitB12 deficiency, requiring further research.

### MMA promotes PanNEN cell proliferation, migration, and invasion

**Fig. 2:**
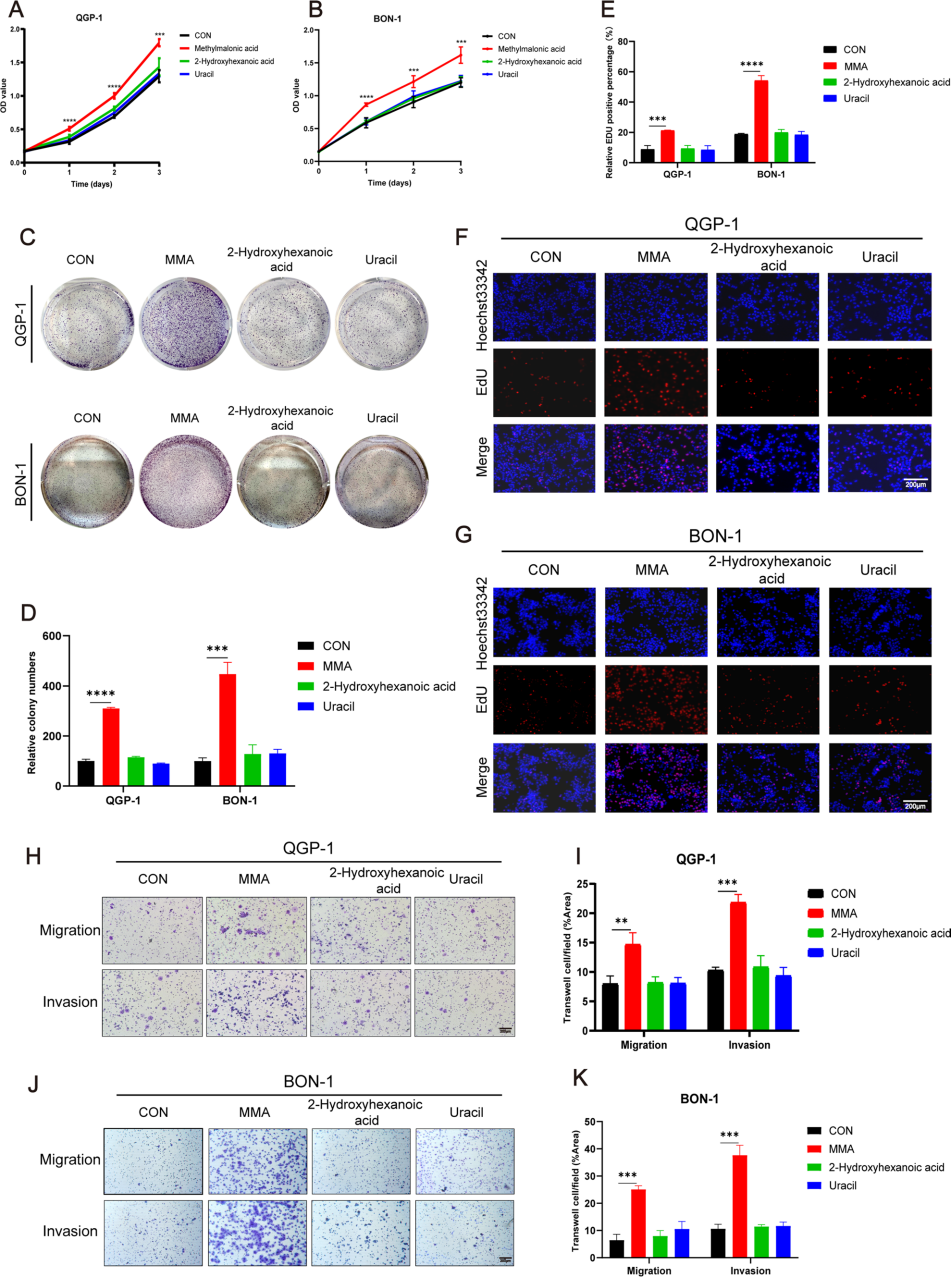
MMA promotes proliferation, migration, and invasion of PanNEN cells. **A–G** The effects of the three significantly elevated metabolites on cell proliferation were tested by cell counting CCK-8 assay (**A, B**), colony formation (**C, D**), and EDU assays (**E–G**) in QGP-1 and BON-1 cells. **H–K** Transwell assays indicated that only MMA significantly increased cell migration and invasion in both QGP-1 (**H**) and BON-1 (**J**) cells compared with the control groups. Statistics of migration and invasion cells in the transwell assays after treatment for 48 h were analyzed (**I, K**)

To determine whether any of these three upregulated metabolites have a role in promoting PanNEN cell progression, we treated QGP-1, BON-1, and IPC619 cells with each metabolite separately. Cell counting CCK-8 assay and colony formation assays demonstrated that MMA was the most significant metabolite promoting PanNEN cell proliferation (Fig. 2A, B; Fig. S3A) and colony formation (Fig. 2C, D; Fig. S3F, H). To further verify the positive influence of MMA on PanNEN cell proliferation, the EDU assay was performed and also showed that cell proliferation was promoted by MMA (Fig. 2E–G; Fig. S3D, E). Besides, transwell assays indicated that MMA played the most remarkable role in promoting the migration and invasion ability of PanNEN cells (Fig. 2H–K; Fig. S3K, M). Moreover, the STC-1 cell lines were used to ascertain the tumor-promoting effect of MMA in small intestinal neuroendocrine tumor cells (Fig. S3).

Several studies have revealed that PanNENs of distant diseases and grade 3 increased with age, and survival of NENs decreased with increasing decade of age, suggesting a correlation between the metastatic rate of PanNENs and aging [30, 31]. Recently, Gomes et al. found that MMA, phosphoenolpyruvate (PEP), and quinolinate (QA) were significantly elevated in elderly serum to be potential oncometabolite for cancer progression. We additionally treated PanNEN cells with MMA, PEP, and QA to evaluate their effects of promoting tumor cell proliferation, migration, and invasion in PanNEN cells, and MMA was identified as the only oncometabolite for PanNEN cells (Fig. S4). Taken together, these results demonstrate that MMA is responsible for promoting PanNEN cell proliferation, migration, and invasion.

### *MMA facilitates EMT *via* increasing the expression of INHBA*

**Fig. 3:**
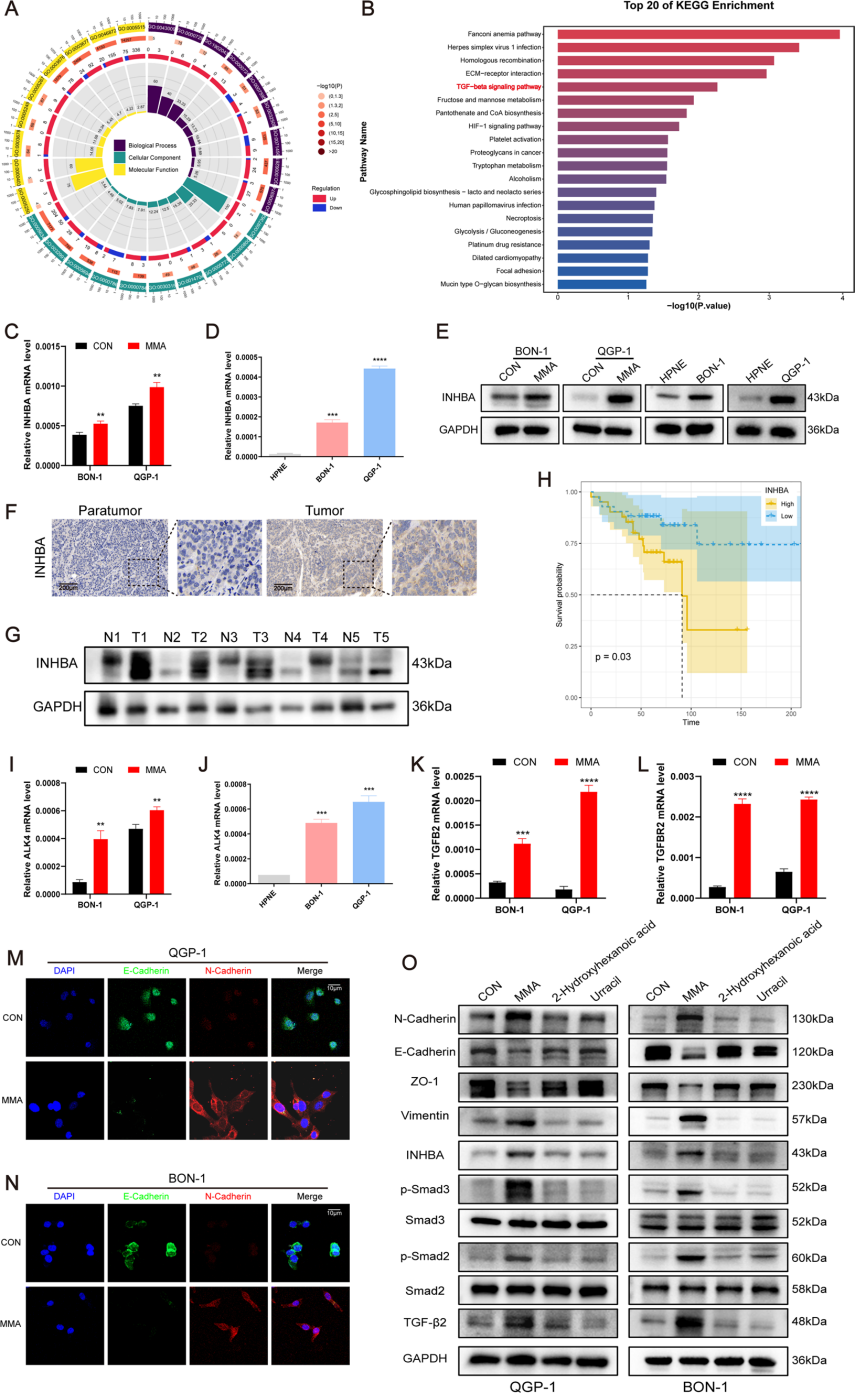
MMA increases the expression of INHBA and EMT markers. **A** GO analysis of RNA seq results showed that MMA might participate in various biological processes. **B** KEGG analyses of RNA‐seq data in MMA-induced and control groups (Top 20 enriched pathways). **C** QPCR was performed to detect the relative expression of INHBA in MMA-treated and the control group in QGP-1 and BON-1 cells. **D** The relative expression of INHBA in human normal pancreatic cells (HPNE) and PanNEN cells (QGP-1 and BON-1) was detected by qPCR assay. **E** The expression of INHBA in indicated groups was evaluated by western blotting. **F** Typical images of INHBA IHC staining in PanNEN tumor tissues and adjacent normal tissues. **G** Western blots indicated that the expression of INHBA was increased in PanNEN tumor tissues compared with that in the adjacent normal tissues of PanNEN patients. *T* tumors; *N* adjacent normal tissues. **H** Patients with high INHBA expression had lower overall survival probabilities in PanNEN patients. **I** The relative expression of ALK4 in MMA-treated and the control group in QGP-1 and BON-1 cells. **J** The relative expression of ALK4 in human normal HPNE and PanNEN cells (QGP-1 and BON-1) was detected by qPCR assay. **K, L** The relative expression of TGFB2 (**K**) and TGFBR2 (**L**) in MMA-treated and the control group in QGP-1 and BON-1 cells. **M, N** Typical IF images of the expression of E-cadherin and N-cadherin for QGP-1 cell lines (**M**) and BON-1 cells (**N**). **O** Western blots showed that only MMA induced EMT and increased the expression of INHBA, TGF-β2, p-Smad2, and p-Smad3 among the three upregulated metabolites

MMA treatment with a concentration of 5 mM for 10 days was determined to be the optimal condition for MMA stimulation of PanNEN cells (Fig. S5). To investigate the molecular mechanism of MMA in PanNEN cells, a global transcriptomic analysis was performed in QGP-1 cells treated with 5 mM MMA for 10 days. GO analysis showed that MMA induced a series of biological processes such as regulation of transcription and signal transduction (Fig. 3A, S6A). KEGG pathway analysis demonstrated that TGF-β signaling pathway was activated in MMA-induced PanNEN cells (Fig. 3B). It is well-known that TGF-β signaling is a common signaling pathway of EMT-mediated tumor metastasis. Among the eight upregulated genes involved in TGF-β pathway induced by MMA, INHBA and INHBB altered largest (Fig. S6B, Supplementary Table 4). The relative INHBA mRNA expression of MMA-induced group increased significantly compared with the control group in QGP-1, BON-1, and IPC619 cells with qPCR validation (Fig. 3C, Fig. S6C). Compared with human normal pancreatic cell lines (HPNE), mRNA expression of INHBA in PanNEN cells was significantly higher (Fig. 3D). Consistent results with the mRNA level were observed at the protein level by western blotting experiments (Fig. 3E, Fig. S6D). To validate the expression of INHBA in PanNEN patients, tumor tissues and adjacent pancreatic tissues from patients with PanNEN were examined via immunohistochemistry staining and western blotting. The results showed that the levels of INHBA in PanNEN tumor tissues were significantly higher compared with adjacent normal tissues (Fig. 3F, G). A bioinformatics analysis based on RNA-seq BAM file EGAS00001005024 was performed to further confirm the relationship between INHBA and clinical characteristics. Patients with high INHBA expression had lower overall survival probabilities in PanNENs (Fig. 3H). Besides, the relative mRNA expression of activin receptors, including ALK4, ACVR2A, and ACVR2B, increased significantly in PanNEN cells compared with HPNE, and MMA-treated PanNEN cells showed higher expression than the control group (Fig. 3I, J; Fig. S6E–H). In addition, the relative TGFB2 and TGFBR2 mRNA expression of MMA-induced group increased significantly compared with the control group in PanNEN cells with qPCR validation (Fig. 3K, L). Furthermore, immunofluorescence analysis showed that the PanNEN cells treated with MMA were characterized by a spindle-shaped morphology and decreased expression of E-cadherin, whereas increased expression of N-cadherin (Fig. 3M, N; Fig. S3J, O). Consistent with these results, Western blotting showed that epithelial markers (E-cadherin, ZO-1) were downregulated, while mesenchymal markers (N-cadherin, vimentin), INHBA, TGF-β2, p-Smad2, and p-Smad3 were upregulated only in MMA-induced PanNEN cells (Fig. 3O; Fig. S3I). However, the expression of INHBB at mRNA and protein levels did not show significant changes (Fig. S6I–K). Overall, our data show that MMA induces the upregulation of INHBA and TGFβ2 and results in EMT in PanNEN cells. We have decided to characterize the role of the INHBA axis in this study, while future studies are needed to delineate the role of the TGFβ2 axis in the future.

### INHBA is required to promote PanNEN cell progression induced by MMA

**Fig. 4:**
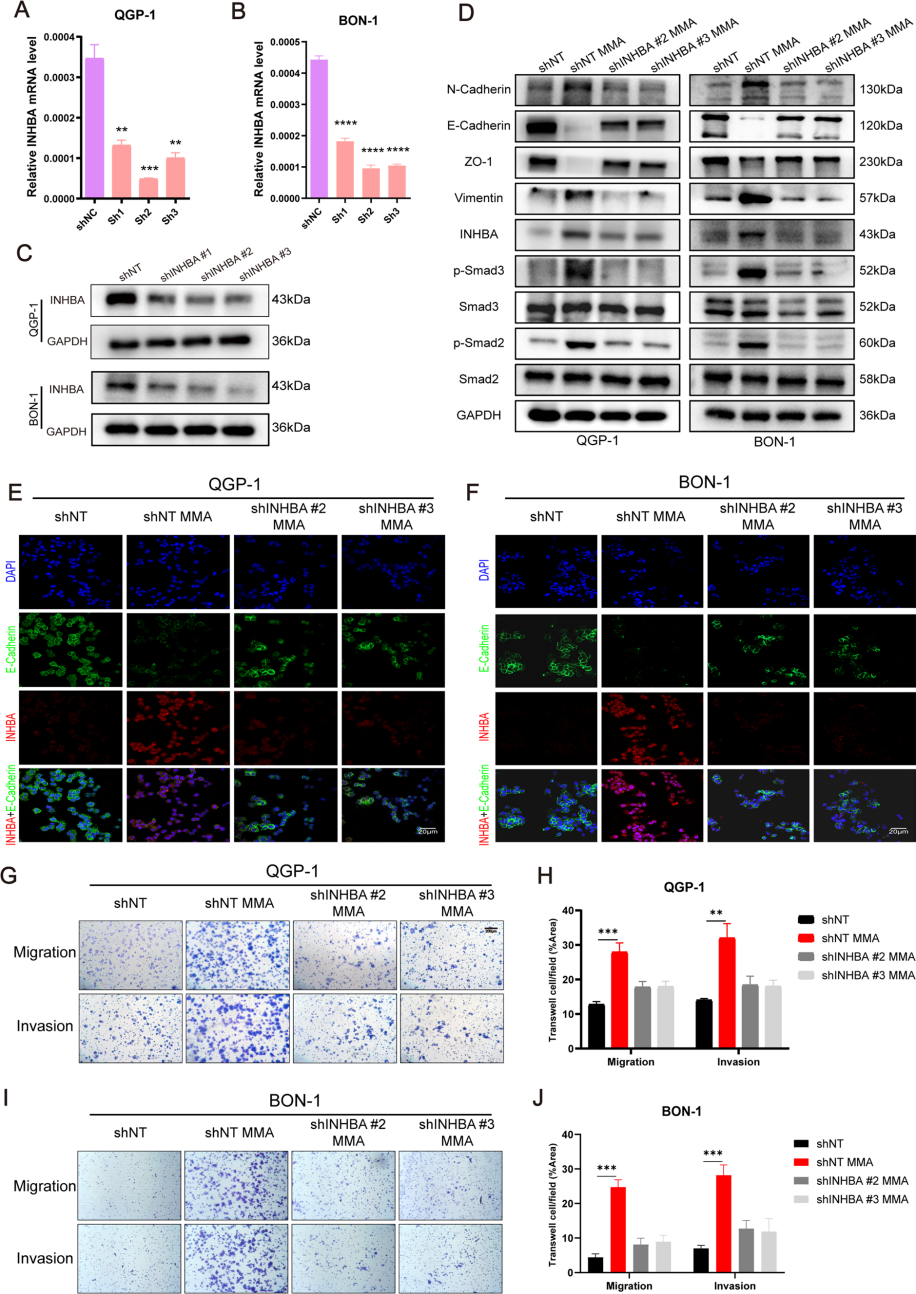
INHBA mediates MMA-induced aggressiveness in PanNEN cells. **A**, **B** INHBA levels in QGP-1 (**A**) and BON-1 cells (**B**) transfected with shRNA lentivirus against INHBA or negative control were evaluated by qPCR. **C** Immunoblots of INHBA expression in QGP-1 and BON-1 cells transfected with shRNA lentivirus against INHBA or negative control. **D** Immunoblots of QGP-1 and BON-1 cells with INHBA knockdown and treated with 5 mM MMA for 10 days. **E–F** Typical IF images of the expression of E-cadherin and INHBA for QGP-1 cell lines (**E**) and BON-1 cells (**F**). **G-J** Transwell migration/invasion assays of QGP-1 (**G, H**) and BON-1 (**I, J**) cells

Next, we examined whether INHBA was required to sustain pro-aggressive effect of MMA in PanNEN cells. To further examine the importance of INHBA, we constructed stably transfected PanNEN cells expressing either non-targeting shRNA control (shNT) or shRNA to INHBA (shINHBA) (Fig. 4A–C; Fig. S7A, B). Among the three shRNAs to INHBA, stably transfected cells expressing shRNA INHBA#2 and shRNA INHBA#3 exhibited the best knockdown and were used for further analysis. The knockdown of INHBA resulted in the elimination of p-Smad2 and p-Smad3 activation and loss of mesenchymal markers (N-cadherin, vimentin) induction, along with reverse of epithelial markers loss (Fig. 4D, Fig. S7C, D). The results of cellular immunofluorescence experiment were consistent with the above results (Fig. 4E, F; Fig. S7E). INHBA knockdown decreased the migrated and invasive ability of PanNEN cells (Fig. S7F–H), and INHBA was required for promotion of cell migration and invasion in MMA-induced PanNEN cells, as assayed in transwell assays (Fig. 4G–J; Fig. S7I, J) and wound healing assays (Fig. S7K–P). In addition, the knockdown of INHBA exhibited negative effects on MMA promoting proliferation and colony formation in PanNEN cells (Fig. S8).

To further determine the function of INHBA, stably transfected cells overexpressing INHBA were constructed (Fig. S9A–C). The transwell assays indicated that overexpression of INHBA increased cell migration and invasion (Fig. S9D–G). Overexpression of INHBA caused loss of epithelial markers, increase of mesenchymal markers, and activation of p-Smad2 and p-Smad3 (Fig. S9H). Besides, time course analysis revealed that MMA induced an increase in INHBA within 24 h, before any of the EMT markers were detected (Fig. S9I), suggesting that the upregulation of INHBA driven by MMA promoted expression of EMT markers in PanNEN cells. Exogenous recombinant human INHBA (rh-INHBA) treatment induced the expression of its own gene (INHBA) along with the loss of epithelial markers and increase of mesenchymal markers (Fig. S9J).

Moreover, signaling antagonist SB431542, which blocks activin type I receptor (ALK4) and TGF-β signaling, was used to demonstrate whether the tumor-promoting effect of MMA could be reversed. Our results indicated that the promotion of MMA in proliferation and colony formation of PanNEN cells was significantly attenuated by SB431542 (Fig. S10A–H). And blockade of INHBA with SB431542 almost eliminated MMA’s promotion of migration, invasion, and EMT (Fig. S10I–M). The same results were observed in the STC-1 cell lines (Fig. S10N–U). Overall, our data show that INHBA is required to promote PanNEN cell proliferation, migration, invasion, and EMT induced by MMA.

### *MMA accelerates PanNEN tumorigenesis and metastasis *via* upregulation of INHBA*

**Fig. 5:**
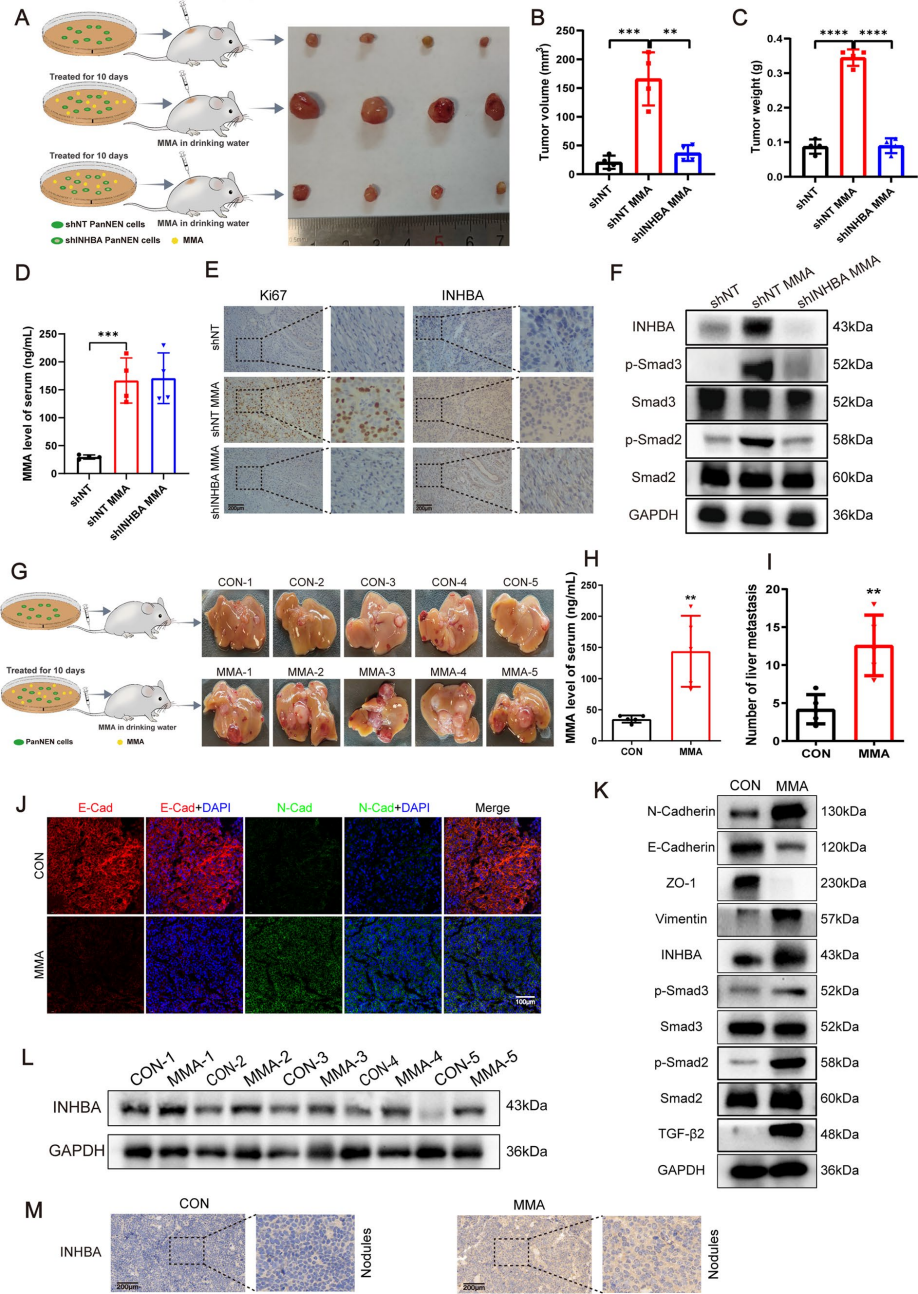
MMA promotes PanNEN progression through INHBA upregulation in vivo. **A** Schematic diagram of the MMA regimen in mice with subcutaneous PanNENs and general view of PanNEN tumorigenesis in nude mice subcutaneously injected with QGP-1 cells treated with ddH_2_O, MMA, and MMA stimulation after INHBA knockdown. *n* = 4 mice per group. **B**, **C** Volume (**B**) and weight (**C**) of subcutaneous tumor of nude mice in three groups. **D** The serum MMA levels of mice after being fed with MMA water compared with the control group. **E** Typical images of IHC staining with Ki67 and INHBA in subcutaneous tumor of nude mice in three groups. **F** Immunoblots of INHBA, p-Smad2, and p-Smad3 expression in subcutaneous tumor of three groups. **G** Schematic diagram of the MMA regimen in mice with tail vein injection and representative images of liver metastases in nude mice 8 weeks after injection through tail vein of MMA-treated and control groups. *n* = 5 mice per group. **H** The serum MMA levels of mice after being fed with MMA water compared with the control group. **I** Number of liver metastasis in MMA-treated and control groups. **J** Typical IF images of the expression of E-cadherin and N-cadherin for liver metastatic nodules of MMA-treated and control groups. **K** Immunoblots of EMT markers, TGF-β2, p-Smad2, and p-Samd3 expression in liver metastatic nodules of MMA-treated and control groups. **L** Immunoblots of INHBA expression in liver metastatic nodules of MMA-treated and control groups. **M** Typical images of INHBA IHC staining in liver metastatic nodules of MMA-treated and control nude mice

Since our in vitro results suggested a mechanistic role of INHBA in MMA-induced tumor promotion, we carried out further investigations in vivo. We explored whether MMA could contribute to PanNEN growth in vivo based on subcutaneous injection of tumor cells. The data indicated that MMA increased the tumor volumes and tumor weights, whereas knocking down INHBA or blockade pathways with SB431542 inhibitor almost abolished the increased tumorigenicity induced by MMA (Fig. 5A–C; Fig. S11A–C). The serum MMA levels of mice after being fed with MMA water were significantly higher than the control group to achieve increased circulatory MMA concentrations and verify the effect of MMA on tumor progression in vivo (Fig. 5D). Subsequently, MMA has also been confirmed in vivo to increase the expression of INHBA, Ki-67, p-Smad2, p-Smad3, and EMT markers, according to immunohistochemistry, immunofluorescence, and western blotting assays of subcutaneous tumor tissues. However, knocking down INHBA or blockade with SB431542 inhibitor almost reversed the expressions of these markers, as expected (Fig. 5E, F; Fig. S11D–F).

We then explored whether MMA could facilitate metastasis of PanNENs in vivo. The animal model of metastatic tumor was established for in vivo investigation by injecting cells treated with MMA or ddH_2_O via tail vein. Liver metastasis was measured (5 mice/group) 8 weeks after the operation (Fig. 5G). The serum MMA levels of mice after being fed with MMA water were significantly higher than the control group (Fig. 5H). Consistent with the results of in vitro experiments, the number of tumor nodules in the liver was increased in mice of MMA-induced group compared with the control group (Fig. 5I). The results of immunofluorescence and western blotting of liver metastatic nodules confirmed that MMA promoted EMT induction of PanNEN cells in vivo (Fig. 5J, K). We also evaluated INHBA expression by immunohistochemical staining and western blotting assays of liver metastatic nodules, and verified that MMA treatment increased the expression of INHBA in vivo (Fig. 5L, M). These results reveal that MMA facilitates tumorigenesis and metastasis of PanNEN via upregulation of INHBA in vivo.

### MMA promotes PanNEN cell progression by FOXA2-initiated INHBA transcription

**Fig. 6:**
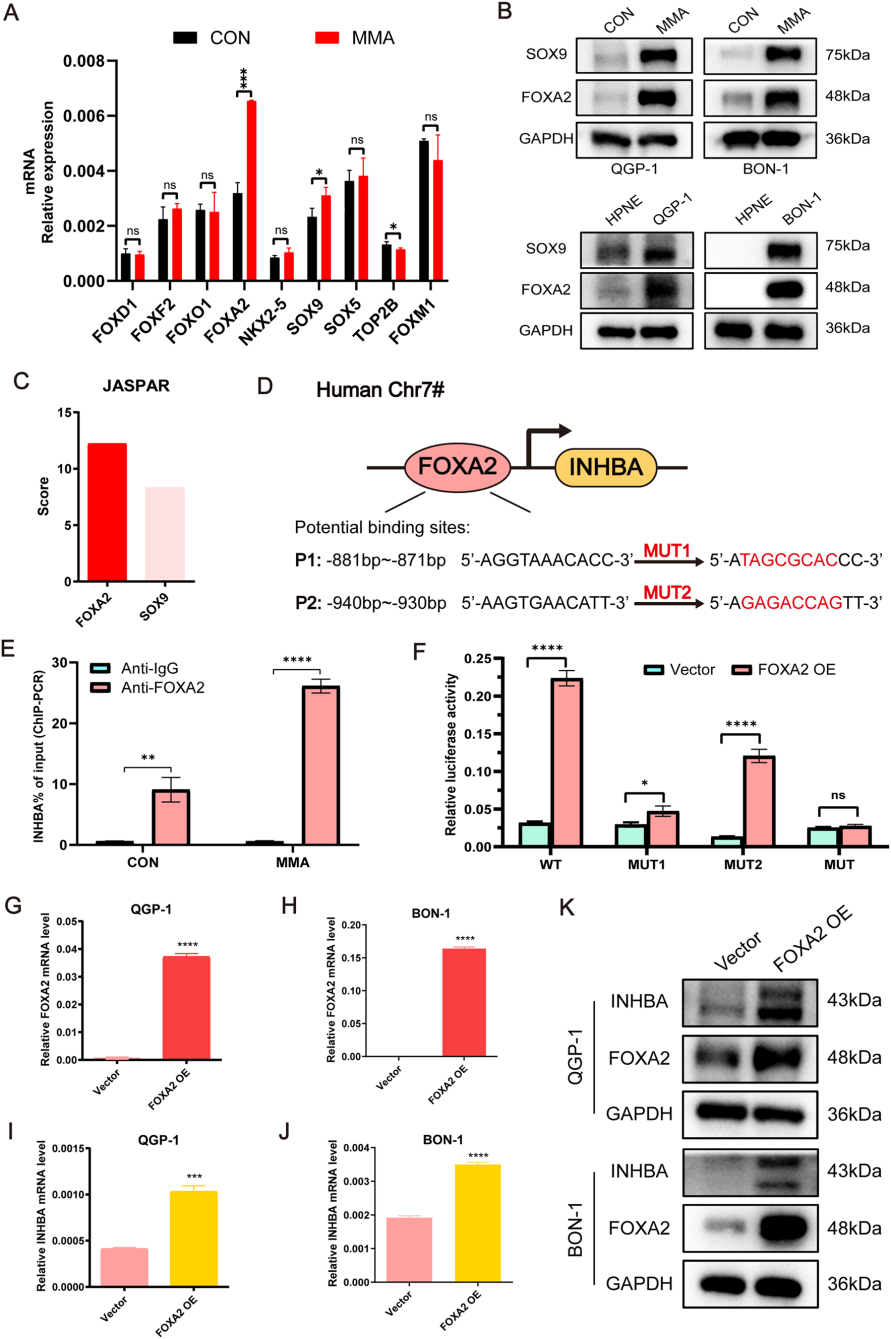
Upregulation of INHBA induced by MMA is the target of transcription factor FOXA2.** A** QPCR was used to evaluate the relative expression of potential transcription factors for INHBA in MMA-induced QGP-1 cells. **B** Immunoblots of SOX9 and FOXA2 expression in indicated groups. **C** The JASPAR score of potential transcription factors, FOXA2 and SOX9, binding to the INHBA promoter region. **D** Schematic diagram demonstrated the potential FOXA2 binding sequence in the INHBA promoter region predicted by JASPAR. **E** ChIP-qPCR analysis of FOXA2 binding to INHBA promoter in QGP-1 cells treated with ddH_2_O and MMA. **F** Dual luciferase reporter assay for detecting the activity of wild-type or mutant INHBA promoters in QGP-1 cells which were transfected with FOXA2 overexpression or vector lentivirus. **G–J** QPCR was performed to detect the efficiency of FOXA2 overexpression and the expression of INHBA in FOXA2 overexpressing groups compared with the control group in QGP-1 (**G**, **I**) and BON-1 cells (**H**, **J**). **K** Western blots indicated that FOXA2 overexpression significantly increased the expression of INHBA in QGP-1 and BON-1 cells

To decipher the transcription factor that may be responsible for the upregulation of INHBA induced by MMA, metascape analysis was performed using the RNA-seq data of MMA-induced QGP-1 cells. A total of nine potential transcription factors driving INHBA expression were identified (Supplementary Table 5), and two of which were significantly increased in MMA-induced PanNEN cells with qPCR and WB validation: FOXA2 and SOX9 (Fig. 6A, B). Besides, FOXA2 and SOX9 expressions were significantly higher in PanNEN cells than normal human pancreatic cells (Fig. 6B; Fig. S12A). Next, the JASPAR CORE database was used to predict the probability of FOXA2 and SOX9 binding to the INHBA promoter region and the potential transcription factor binding sites (TFBS). The JASPAR scores indicated that FOXA2 was more likely to bind to the INHBA promoter region than SOX9 (Fig. 6C). In addition, FOXA2 and SOX9 were used as transcription factors to predict the potential downstream target genes in reverse via hTFtarget, and it was found that INHBA was one of the potential target genes of FOXA2, while the potential downstream target genes of SOX9 did not contain INHBA (Supplementary Table 5). Thus, FOXA2 was characterized to be putative upstream transcription factor targeting INHBA for further investigation.

The prediction of FOXA2 binding sites in the INHBA promoter region revealed two potential motifs (P1, P2) with high scores (Fig. 6D). Chromatin immunoprecipitation analysis confirmed that FOXA2 was recruited to the INHBA promoter region and MMA increased the binding of FOXA2 to the INHBA promoter by threefold (Fig. 6E). Further, luciferase reporter gene plasmids containing the wild type (WT), P1 mutant (MUT1), P2 mutant (MUT2), and combined mutant sites (MUT) were cloned into pGL3.0 vector. Our data revealed that overexpression of FOXA2 increased the WT, MUT1 or MUT2 reporter activity; however, the luciferase activity of the MUT was not affected and the reporter activity of MUT1 and MUT2 with FOXA2 OE was lower than WT (Fig. 6F). These data suggested that both the sites P1 and P2 of INHBA promoter were the binding regions of FOXA2.

**Fig. 7:**
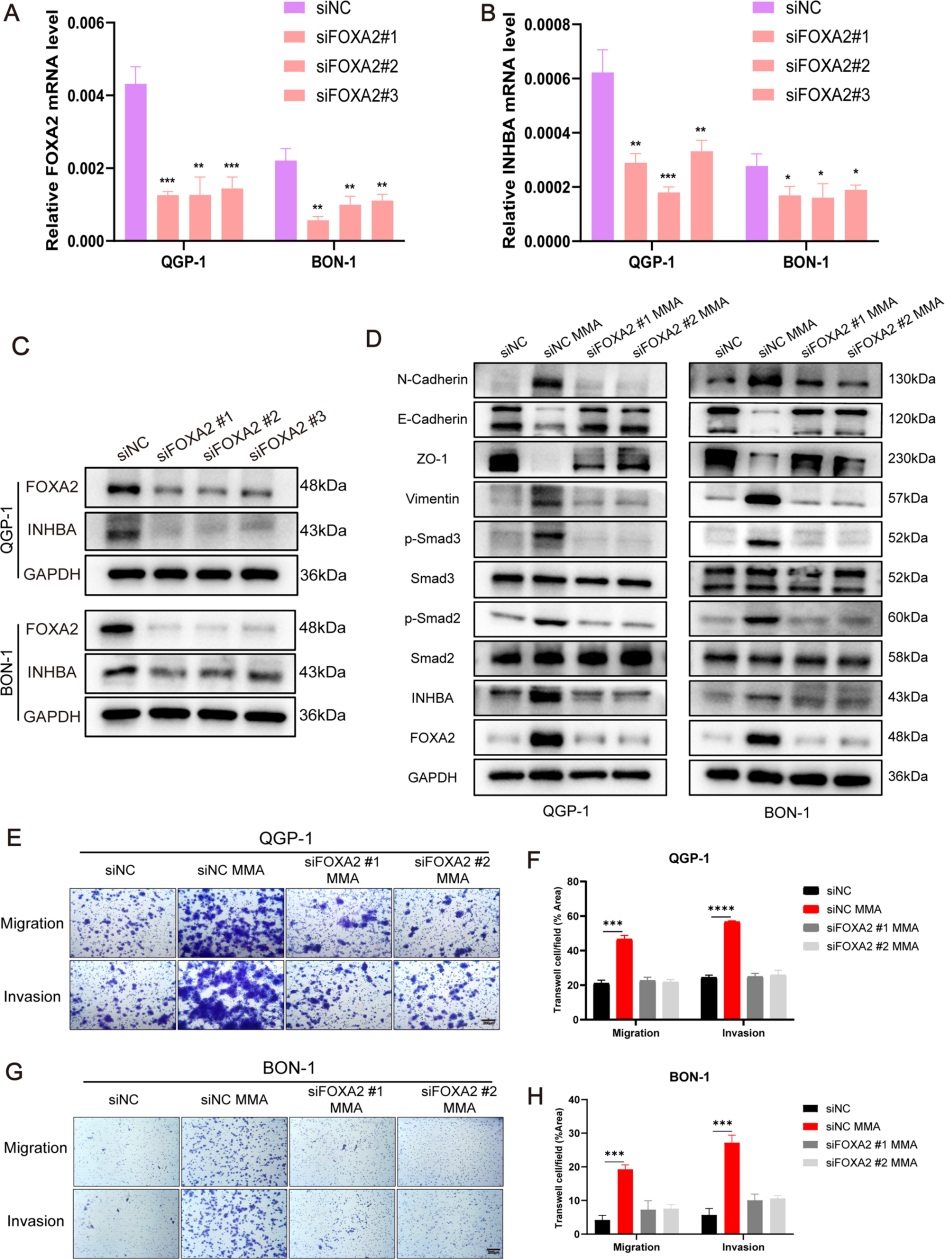
FOXA2 knockdown decreases INHBA expression and MMA/INHBA-mediated aggressiveness in PanNEN cells. **A**, **B** FOXA2 and INHBA levels in QGP-1 and BON-1 cells transfected with FOXA2 siRNA or Scramble siRNA were evaluated by qPCR. **C** Immunoblots of FOXA2 and INHBA expression in QGP-1 and BON-1 cells transfected with FOXA2 siRNA or Scramble siRNA. **D** Immunoblots of QGP-1 and BON-1 cells with FOXA2 knockdown and treated with 5 mM MMA for 10 days. **E–H** Transwell migration/invasion assays of QGP-1 (**E**, **F**) and BON-1 (**G**, **H**) cells

Next, we sought to further verify the regulatory role of transcription factor FOXA2 on INHBA. Our results showed that overexpression of FOXA2 significantly increased INHBA expression at both transcriptional and translational levels (Fig. 6G–K; Fig. S12B–D). Analogously, knockdown of FOXA2 using small interfering RNA (siRNA) remarkably reduced INHBA expression at both transcriptional and translational levels (Fig. 7A–C; Fig. S12E–G), and attenuated MMA-induced EMT induction (Fig. 7D; Fig. S12J). Meanwhile, transwell assays revealed that FOXA2 silencing could remarkably impede the promotion of cell migration and invasion induced by MMA (Fig. 7E–H; Fig. S12H, I). These results elucidate that MMA promotes PanNEN cell progression by FOXA2-initiated INHBA transcription.

### INHBA upregulation induced by MMA activates MITF to regulate EMT

**Fig. 8:**
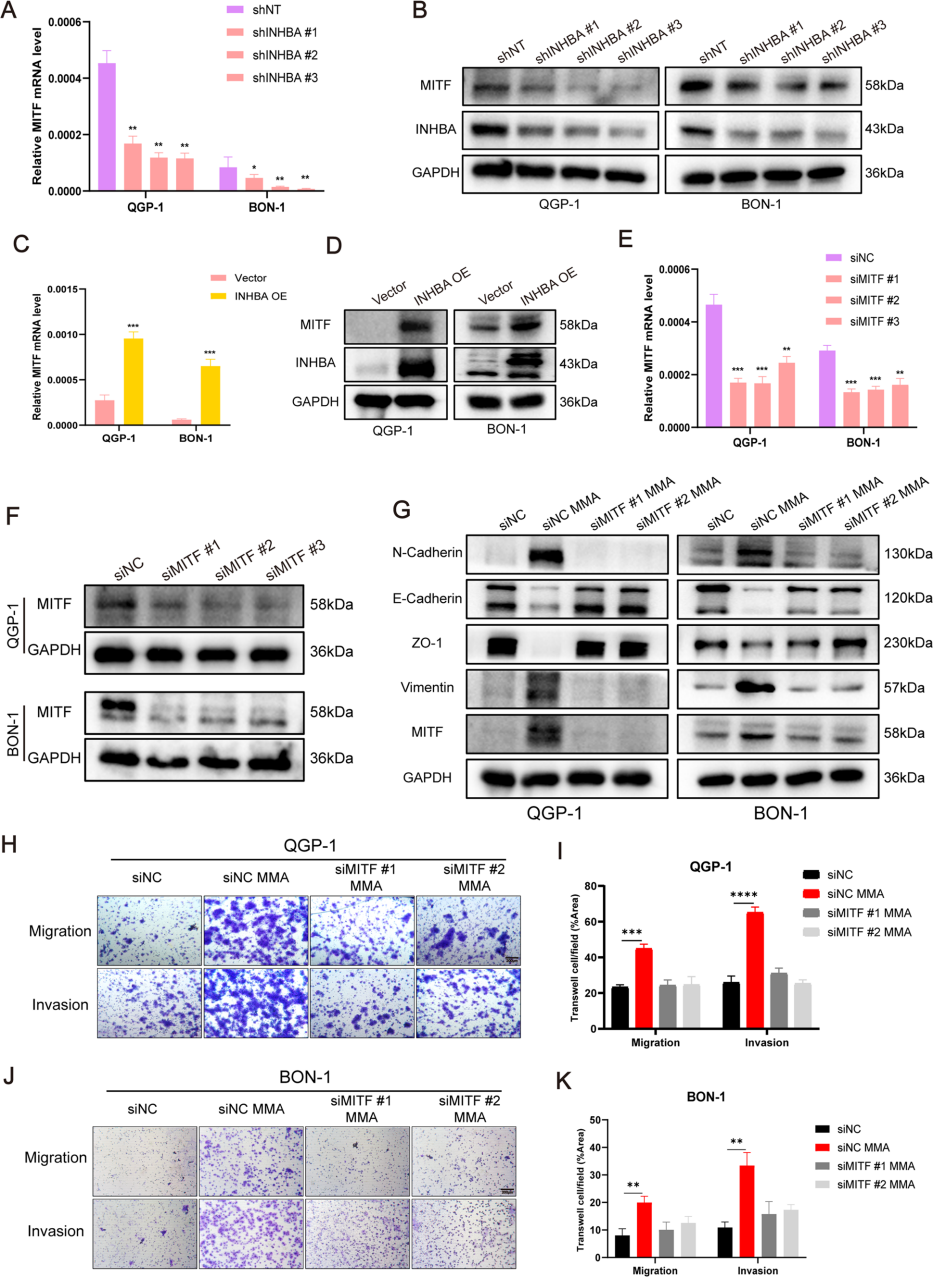
INHBA upregulation by MMA induces MITF-mediated EMT, migration, and invasion in PanNEN cells.** A** MITF levels in QGP-1 and BON-1 cells transfected with INHBA shRNA or Scramble shRNA were evaluated by qPCR. **B** Immunoblots of INHBA and MITF expression in QGP-1 and BON-1 cells transfected with INHBA shRNA or Scramble shRNA. **C** MITF expression was evaluated in QGP-1 and BON-1 cells transfected with INHBA overexpression or vector lentivirus by qPCR. **D** Western blots indicated that INHBA overexpression significantly increased the expression of MITF in QGP-1 and BON-1 cells. **E** MITF levels in QGP-1 and BON-1 cells transfected with MITF siRNA or Scramble siRNA were evaluated by qPCR. **F** Immunoblots of MITF expression in QGP-1 and BON-1 cells transfected with MITF siRNA or Scramble siRNA. **G** Immunoblots of QGP-1 and BON-1 cells with MITF knockdown and treated with 5 mM MMA for 10 days. **H**–**K** Transwell migration/invasion assays of QGP-1 (**H**, **I**) and BON-1 (**J**, **K**) cells

It is known that INHBA, as a secreted protein, binds to activin receptors and phosphorylates Smad2 and Smad3. Then the active transcription complex translocates into the nucleus, binding to a specific SBE sequence and co-regulates the expression of target genes with DNA binding transcription factors [32]. To demonstrate how INHBA promotes EMT-mediated tumor metastasis and identify the potential downstream molecules of INHBA, RNA seq of QGP-1 overexpressing INHBA and control QGP-1 cells was performed. There were 623 genes differentially expressed, including 40 transcription factors, among which 17 transcription factors were upregulated in INHBA overexpressing group (Supplementary Table 6). One of the most upregulated of these transcription factors was microphthalmia-associated transcription factor (MITF), which has been verified as a regulator of EMT. The knockdown of INHBA by shRNA resulted in remarkable reduction of MITF expression at both transcriptional and translational levels (Fig. 8A, B; Fig. S12K, L). Meanwhile, stably transfected cells overexpressing INHBA significantly increased MITF expression by qPCR and western blotting validation (Fig. 8C, D; Fig. S12M, N). Then we used siRNA to suppress MITF expression in PanNEN cells to further verify the role of MITF on EMT (Fig. 8E, F; Fig. S12O,P). Upon MITF suppression, treatment with MMA failed to upregulate the EMT markers (Fig. 8G; Fig. S12Q). Moreover, suppression of MITF almost reversed the ability of MMA to promote migratory and invasive properties in PanNEN cells (Fig. 8H–K; Fig.S12R,S). Overall, the upregulation of INHBA by MMA activates MITF to regulate EMT induction in PanNEN cells.

## Discussion

Metastasis represents the primary cause of tumor-associated death in patients with PanNEN [33]. Accumulating evidence shows that alterations in tumor cell metabolism are emerging as key factors governing tumor metastasis [24, 34]. The enhanced understanding of the metabolic dysregulation underlying the metastasis process might provide innovative therapeutic targets for PanNEN therapy. In this study, based on serum metabolomics, MMA was considered as a potential oncometabolite for the metastasis of PanNENs for the first time, which has been validated in vitro and in vivo. One of the key findings was the potentially novel mechanism of EMT triggered by MMA accumulation. We demonstrated that the upregulation of INHBA initiated by FOXA2 promoted MITF-mediated EMT and tumor progression in response to sustained MMA stimulation. These results put forward a novel therapeutic target for PanNENs.

Several clinical studies have reported the stratified metabolic phenotype of various neuroendocrine neoplasmas to identify novel biomarkers for diagnosis and prognosis based on metabolic profiling of urine samples [35], tissue samples [36], and plasma samples [37]. It has been revealed that cell metabolism possessed heterogeneous characteristics and varied depending on the site and origin of tumors [15]. To characterize the impact of metabolites on PanNEN metastasis, we performed metabolomics analysis of serum from PanNEN patients with or without metastasis to recognize the upregulated circulating metabolites that might be associated with PanNEN metastasis. Ultimately, MMA was characterized as a serum oncometabolite responsible for PanNEN progression for the first time.

MMA is a by-product of the enzymatic reactions leading to conversion of propionyl-CoA to succinyl-CoA, which can fuel the tricarboxylic acid (TCA) cycle [38]. Recently, Gomes et al. have identified three upregulated metabolites (phosphoenolpyruvate, quinolinate, and MMA) in the aged serum, and MMA was verified as an oncometabolite to increase cancer progression in breast and lung cancers [28]. And their subsequent study showed that cancer cells themselves could increase MMA levels by altering propanoate metabolism to promote cancer progression in breast and lung cancers [39]. We compared the ages of PanNEN patients in the metastatic and non-metastatic groups in this study and found no significant differences between the two groups. We also stimulated PanNEN cells with the three elevated metabolites of the aged serum to verify their effects on PanNEN cells, and the results indicated that only MMA could promote the proliferation, migration, and invasion of PanNEN cells, which was consistent with Gomes’ and our results. Although MMA was identified as oncometabolite to promote tumor progression both in Gomes’ and our study based on serum metabolomics of different groups of patients, we found that the specific molecular mechanism of MMA in promoting the progression in PanNEN patients was different from that in breast and lung cancers of Gomes’ study, which might be related to the heterogeneity of the oncogenic function of metabolites in tumors of different origins and sites [15]. Our findings implicated MMA as an oncometabolite of PanNEN progression and innovatively established INHBA as a critical regulator of the tumor progression in PanNENs induced by MMA.

INHBA is composed of an α subunit and a βA subunit, which forms a disulfide-linked homodimer called activin A [40]. As a secretory protein, INHBA is a member of the TGF-β superfamily and shares the Smad intracellular signaling proteins with TGF-β [41]. The signaling pathway is triggered by activin A binding to activin type II receptors, ActR-IIA and ActR-IIB, then recruiting, binding, and phosphorylating the type I receptor ActR-IB, known as activin receptor-like kinase 4 (ALK4), resulting in the activation of Smad-dependent signaling downstream. The activated Smad complex then translocates and accumulates in the nucleus to participate in the transcriptional regulation of target genes [32]. Emerging evidence has demonstrated that INHBA serves important roles in a wide variety of cancer progressions [42]. However, there is no report on the regulation of INHBA and its role in PanNEN progression. In this study, we first identified INHBA as the vital regulator of MMA-induced PanNEN progression and validated in vitro and in vivo. Furthermore, we found that phosphorylation of Smad3 was involved in INHBA-mediated EMT. To better elucidate the induction of EMT by INHBA in PanNENs, we defined 17 upregulated transcription factors in INHBA-overexpressed PanNEN cells and further analyses identified MITF as a regulator of EMT downstream of INHBA, which has been demonstrated in various aggressive cancers [43].

Analysis of MMA-induced QGP-1 cells based on Metascape and JASPAR databases revealed that FOXA2 might be a potential transcription factor regulating INHBA transcriptional activation in MMA-induced PanNEN cells. INHBA was validated to be a target of the transcription factor FOXA2, according to ChIP data and dual-luciferase reporter analysis. FOXA2, the NE-specific transcription factor, is known to be expressed continuously in all pancreatic cell types during pancreatic development and peak in pancreatic islets [44]. Although FOXA2 was regarded to suppress EMT during pancreatic ductal adenocarcinoma malignant progression [45], genomic analyses depicted that pancreatic progenitor tumors preferentially express genes involved in early pancreatic development (FOXA2) [46]. Notably, FOXA2 was critical to drive the formation of neuroendocrine phenotype and neuroendocrine prostate tumors [47]. Therefore, these results implicated that FOXA2 was a NE-specific transcription factor activating INHBA transcription to promote PanNEN progression, which might be a potential therapeutic target for PanNENs. But how MMA activates the expression of FOXA2 is still unclear, which needs future research in the future.

However, this study suffered from several limitations. First, due to the rarity of PanNENs and the fact that the majority of patients have already exhibited metastases at the time of diagnosis, few serum samples from patients with non-metastatic PanNEN were available, which seemed to be inadequate to reach greater reliability. However, the differential metabolite MMA identified based on the serum metabolomics has been indeed confirmed to be an oncometabolite in this study and Gomes' [28, 39]. Further multi-centric and large-scale studies of PanNENs are required to identify more oncometabolites. Second, the role and mechanisms of MMA need to be explored in more tumors in the future, since the functions of metabolites vary in various tumors depending on different origins and sites. And the role of the TGF-β2 pathway was not further studied in this study and warrants future investigation. Third, recent study has revealed that MMA also activated fibroblasts in the tumor microenvironment (TME) to secrete IL-6, driving cancer progression, drug resistance, and metastasis in breast and lung cancers [48]. A further question raised is that whether MMA impacts other cells in the tumor microenvironment, such as fibroblasts, adipocytes, and immune cells in PanNENs, which provides opportunities for future research. Nonetheless, this study first puts forward the role of oncometabolite on neuroendocrine neoplasm, broadening new horizons in the mechanism study in neuroendocrine neoplasm progression and providing novel potential therapeutic targets.

## Conclusion

In conclusion, this study found that the previously unappreciated oncometabolite MMA increased the expression of INHBA by the neuroendocrine-specific transcription factor, FOXA2, to induce MITF-mediated EMT during the progression of PanNENs, providing an actionable therapeutic vulnerability to metabolic therapy in PanNENs.

### Supplementary Information

Below is the link to the electronic supplementary material.

Supplementary Figure 1 Construction and validation of primary IPC619 cells isolated from neuroendocrine neoplasm tissues of a female patient diagnosed with non-functional-PanNET. **A** Typical images of immunofluorescence with neuroendocrine (NE) biomarkers in primary IPC619 cells. **B** Immunoblots of NE biomarkers expression in primary IPC619 cells.Supplementary file1 (TIF 14796 KB)

Supplementary Figure 2 Comparison of the renal function indicators and vitamin B12 (VitB12) in samples from metastatic (M) and non-metastatic PanNENs (NM). **A**–**C** Comparison of the renal function indicators of patients, including estimated glomerular filtration rate (eGFR, **A**), blood creatinine (Cr, **B**) and blood urea nitrogen (BUN, **C**). **D** Comparison of serum VitB12 concentrations in samples from metastatic (M) and non-metastatic PanNEN (NM).Supplementary file2 (TIF 7401 KB)

Supplementary Figure 3 MMA promotes proliferation and EMT mediated migration and invasion of neuroendocrine tumor cells. **A**–**H** The effects of the three significantly elevated metabolites on cell proliferation were tested by cell counting CCK-8 (**A**, **B**), EDU assays (**C**–**E**), and colony formation (**F**–**H**) in IPC619 and STC-1 cells.** I **Western blots showed that only MMA induced EMT and increased the expression of INHBA, p-Smad2 and p-Smad3 among the three upregulated metabolites in IPC619 and STC-1 cells. **J** Typical IF images of the expression of E-cadherin and N-cadherin for IPC619 cells. **K**–**N** Transwell assays indicated that only MMA significantly increased cell migration and invasion in both IPC619 (**K**) and STC-1 (**L**) cells compared with the control groups. Statistics of migration and invasion cells in the transwell assays after treatment for 48 h were analysed (**M**, **N**). **O** Typical brightfield images to show changes in the morphology for QGP-1 cells induced by MMA compared with control.Supplementary file3 (TIF 32164 KB)

Supplementary Figure 4 Analysis of PanNEN cells treated with the three upregulated metabolites of aged serum. **A** There was no significant difference in ages of metastatic and non-metastatic PanNEN patients. **B**–**J** The effects of the three significantly upregulated metabolites of aged serum on cell proliferation were tested by cell counting CCK-8 (**B**–**D**), EDU assays (**E**–**H**) and colony formation (**I**, **J**) in QGP-1, BON-1 and IPC619 cells. **K** Immunoblots of EMT markers in QGP-1, BON-1 and IPC619 cells treated with the three metabolites. **L**–**Q** Transwell assays indicated that only MMA significantly increased cell migration and invasion in QGP-1 (**L**), BON-1 (**M**) and IPC619 (**N**) cells instead of phosphoenolpyruvate (PEP) or quinolinate (QA). Statistics of migration and invasion cells in the transwell assays after treatment for 48 h were analysed (**O**–**Q**). Supplementary file4 (TIF 14996 KB)

Supplementary Figure 5 Determination of optimal conditions for MMA stimulation of PanNEN cells. **A** Immunoblots of EMT markers in QGP-1, BON-1 and IPC619 cells treated with indicated concentrations of MMA. **B** Immunoblots of EMT markers in QGP-1, BON-1 and IPC619 cells treated with 5mM MMA for indicated time. Supplementary file5 (TIF 25364 KB)

Supplementary Figure 6 RNA seq analysis on MMA-induced QGP-1 cells. **A** GO analysis showed that MMA induced a series of biological processes in QGP-1 cells. **B** There were 8 up-regulated genes enriched in TGF-β pathway in MMA-induced QGP-1 cells. **C** QPCR was performed to detect the relative expression of INHBA in MMA treated and the control group in IPC619 cells. **D** The expression of INHBA in indicated groups were evaluated by western blotting. **E**, **F** The relative expression of ACVR2A (**E**) and ACVR2B (**F**) in human normal HPNE and PanNEN cells (QGP-1 and BON-1) was detected by qPCR assay. **G**, **H** The relative expression of ACVR2A (**G**) and ACVR2B (**H**) in MMA treated and the control group in QGP-1 and BON-1 cells. **I** The relative expression of INHBB in human normal HPNE and PanNEN cells (QGP-1, BON-1 and IPC619) was detected by qPCR assay. **J** QPCR was performed to detect the relative expression of INHBB in MMA treated and the control group in QGP-1, BON-1 and IPC619 cells. **K** Immunoblots of INHBB expression in indicated groups. Supplementary file6 (TIF 27152 KB)

Supplementary Figure 7 INHBA is required for MMA to promote PanNEN cell progression. **A** INHBA levels in IPC619 cells transfected with shRNA lentivirus against INHBA or negative control were evaluated by qPCR. **B** Immunoblots of INHBA expression in IPC619 cells transfected with shRNA lentivirus against INHBA or negative control. **C** Immunoblots of EMT-related markers, p-Smad2 and p-Smad3 in QGP-1 and BON-1 cells transfected with shRNA lentivirus against INHBA or negative control. **D** Immunoblots of IPC619 cells with INHBA knockdown and treated with 5 mM MMA for 10 days. **E** Typical IF images of the expression of E-cadherin and INHBA for IPC619 cells. **F**–**J** Transwell migration/invasion assays of PanNEN cells. INHBA knockdown decreased the migrated and invasive ability of QGP-1 and BON-1 cells (**F**–**H**). INHBA was required for promotion of cell migration and invasion in MMA-induced IPC619 cells (**I**, **J**). **K**–**P** Wound healing assays showed that MMA induced cell migration was attenuated by the knockdown of INHBA in QGP-1, BON-1 and IPC619 cells. Supplementary file7 (TIF 43631 KB)

Supplementary Figure 8 INHBA knockdown decreases the MMA induced proliferation in PanNEN cells. Knockdown of INHBA with shRNA attenuated the role of MMA on proliferation detected by cell counting CCK-8 (**A**–**C**), EDU assays (**D**–**G**) and colony formation (**H**, **I**) in QGP-1, BON-1 and IPC619. Supplementary file8 (TIF 23863 KB)

Supplementary Figure 9 Increased INHBA expression promotes PanNEN cell progression. **A**–**C** QPCR was performed to detect the efficiency of INHBA overexpression in QGP-1 (**A**), BON-1 (**B**) and IPC619 cells (**C**). **D** Overexpression of INHBA significantly increased cell migration and invasion in QGP-1, BON-1 and IPC619 cells when compared with the control groups. **E**–**G** Statistics of migration and invasion cells of the transwell assay after treatment for 48 h in QGP-1 (**E**), BON-1 (**F**) and IPC619 cells (**G**). **H** Western blots indicated that INHBA overexpression significantly increased the EMT markers, p-Smad2 and p-Smad3 in QGP-1, BON-1 and IPC619 cells. **I **Immunoblots of INHBA expression in QGP-1, BON-1 and IPC619 cells treated with MMA for indicated time. **J** Immunoblots of INHBA and EMT markers expression in QGP-1, BON-1 and IPC619 cells treated with recombinant human INHBA with indicated concentrations. Supplementary file9 (TIF 38785 KB)

Supplementary Figure 10 Inhibitor SB431542 attenuates the MMA induced proliferation, migration and invasion of NEN cells. **A**–**H** Activin A signaling antagonist SB431542 decreased the role of MMA on proliferation detected by cell counting CCK-8 (**A**–**C**), colony formation (**D**,**E**), and EDU assays (**F**–**H**) in QGP-1, BON-1 and IPC619 cells. **I** Immunoblots of QGP-1, BON-1 and IPC619 cells with SB431542 and treated with 5 mM MMA for 10 days. **J**–**M** Transwell assays indicated that SB431542 attenuated MMA induced cell migration and invasion in QGP-1, BON-1 and IPC619 cells (**J**). Statistics of migration and invasion cells in the transwell assays after treatment for 48 h were analysed (**K**–**M**). **N**–**R** Inhibitor SB431542 decreased the role of MMA on proliferation detected by cell counting CCK-8 (**N**), EDU assays (**O**, **P**) and colony formation (**Q**, **R**) in STC-1 cells. **S**, **T** Transwell assays indicated that SB431542 attenuated MMA induced cell migration and invasion in STC-1 cells (**S**). Statistics of migration and invasion cells in the transwell assays after treatment for 48 h were analysed (**T**). **U** Immunoblots of STC-1 cells with SB431542 and treated with 5 mM MMA for 10 days. Supplementary file10 (TIF 49835 KB)

Supplementary Figure 11 Inhibitor SB431542 attenuates tumorigenesis induced by MMA in mice. **A** Schematic diagram of the MMA regimen in mice with subcutaneous PanNENs and general view of PanNEN tumorigenesis in nude mice subcutaneously injected with QGP-1 cells treated with ddH2O, MMA and MMA stimulation combined with SB431542. *n* = 5 mice per group. **B**–**C** Weight (**B**) and volume (**C**) of subcutaneous tumor of nude mice in three groups. **D** Typical images of IHC staining with Ki67 and INHBA in subcutaneous tumor of nude mice in three groups. **E** Typical IF images of the expression of E-cadherin and N-cadherin in subcutaneous tumor in three groups. **F** Immunoblots of EMT markers, INHBA, p-Smad2 and p-Smad3 expression in subcutaneous tumor of three groups. Supplementary file11 (TIF 32472 KB)

Supplementary Figure 12 MMA induces EMT in PanNEN cells by activating the FOXA2-INHBA-MITF axis. **A** Immunoblots of SOX9 and FOXA2 expression in indicated groups. **B**, **C** QPCR was performed to detect the efficiency of FOXA2 overexpression and the expression of INHBA in FOXA2 overexpressing groups compared with the control group in IPC619 cells. **D** Western blots indicated that FOXA2 overexpression significantly increased the expression of INHBA in IPC619 cells. **E**, **F** FOXA2 and INHBA levels in IPC619 cells transfected with FOXA2 siRNA or Scramble siRNA were evaluated by qPCR. **G** Immunoblots of FOXA2 and INHBA expression in IPC619 cells transfected with FOXA2 siRNA or Scramble siRNA. **H**, **I** Transwell migration/invasion assays of IPC619 cells. **J** Immunoblots of IPC619 cells with FOXA2 knockdown and treated with 5 mM MMA for 10 days. **K** MITF levels in IPC619 cells transfected with INHBA shRNA or Scramble shRNA were evaluated by qPCR. **L** Immunoblots of INHBA and MITF expression in IPC619 cells transfected with INHBA shRNA or Scramble shRNA. **M** MITF expression was evaluated in IPC619 cells transfected with INHBA overexpression or vector lentivirus by qPCR. **N** Western blots indicated that INHBA overexpression significantly increased the expression of MITF in IPC619 cells. **O** Immunoblots of MITF expression in IPC619 cells transfected with MITF siRNA or Scramble siRNA. **P** MITF levels in IPC619 cells transfected with MITF siRNA or Scramble siRNA were evaluated by qPCR. **Q** Immunoblots of IPC619 cells with MITF knockdown and treated with 5 mM MMA for 10 days. **R**, **S** Transwell migration/invasion assays of IPC619 cells. Supplementary file12 (TIF 31166 KB)

Supplementary file13 (DOCX 18 KB)

Supplementary file14 (RAR 23669 KB)

## Data Availability

All data generated or analyzed during this study are included in this published article and its supplementary information files.
