# Supplementary figures and images for: FOXA2-initiated transcriptional activation of INHBA induced by methylmalonic acid promotes pancreatic neuroendocrine neoplasm progression
Source: Cell Mol Life Sci. 2024 Jan 22;81(1):50. doi: 10.1007/s00018-023-05084-0 (PMC10803496; doi:10.1007/s00018-023-05084-0)

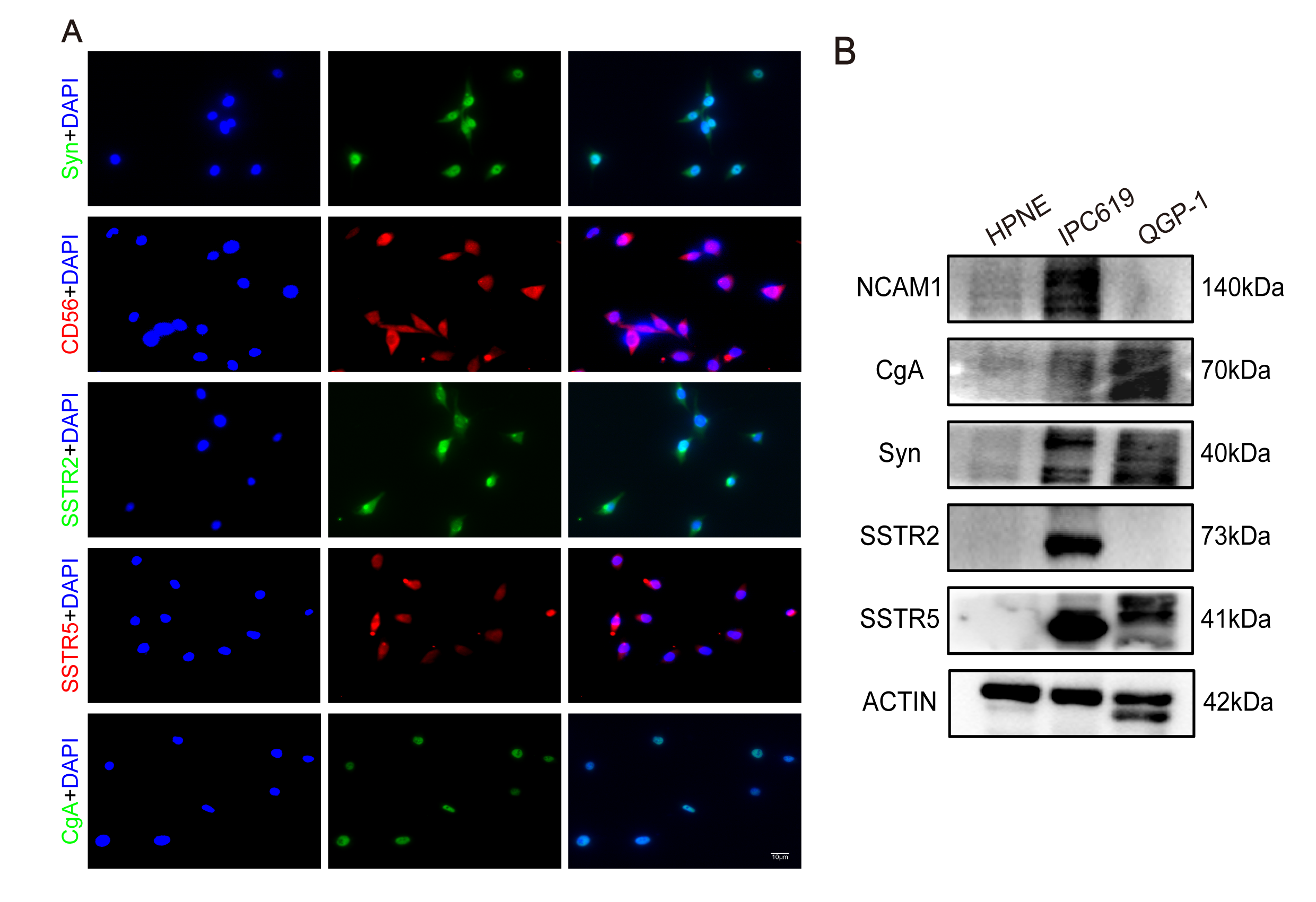

Supplement: Supplementary file 1 — Construction and validation of primary IPC619 cells isolated from neuroendocrine neoplasm tissues of a female patient diagnosed with non-functional-PanNET. A Typical images of immunofluorescence with neuroendocrine (NE) biomarkers in primary IPC619 cells. B Immunoblots of NE biomarkers expression in primary IPC619 cells.Supplementary file1 (TIF 14796 KB) [file 18_2023_5084_MOESM1_ESM.tif]

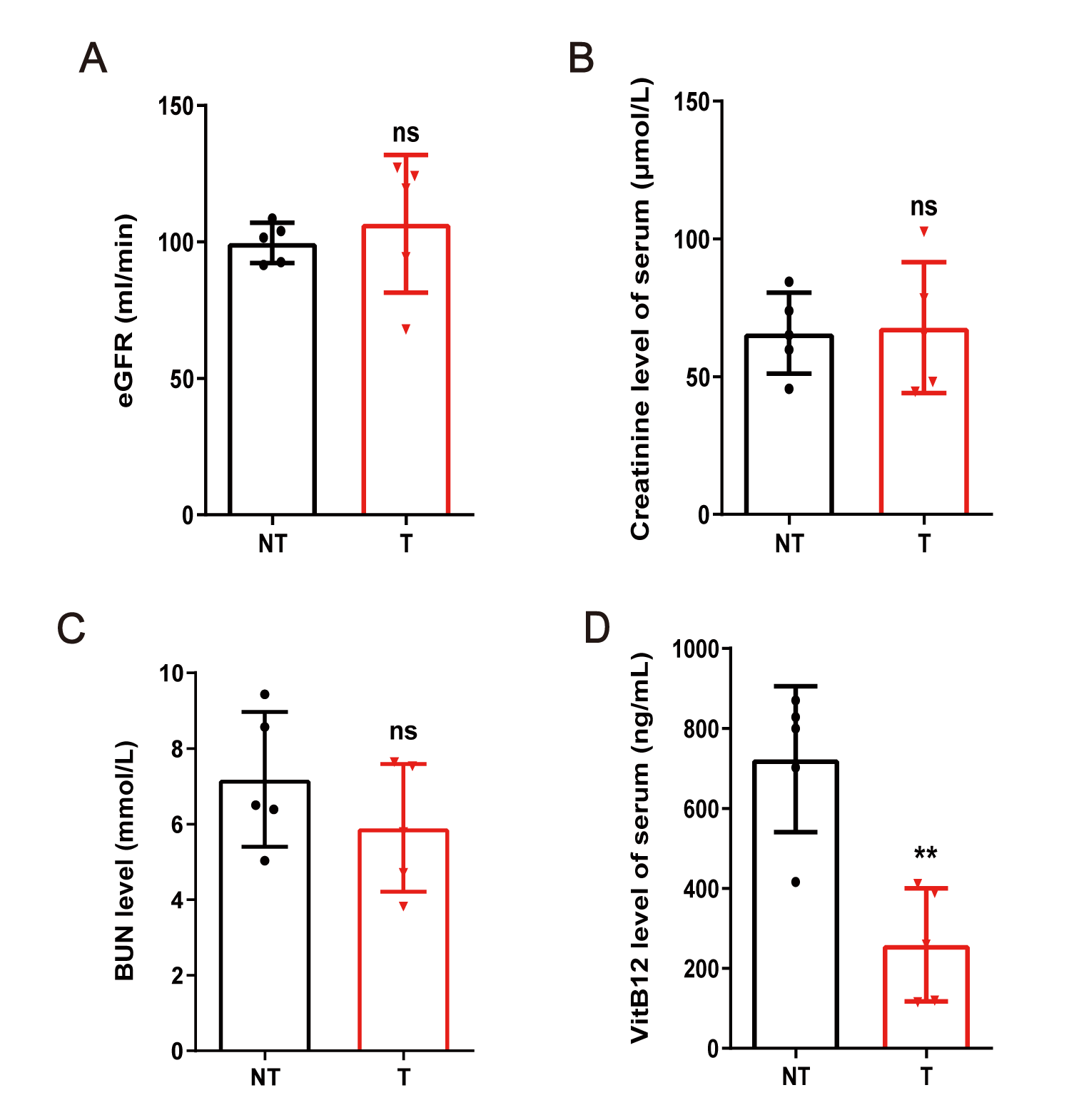

Supplement: Supplementary file 2 — Comparison of the renal function indicators and vitamin B12 (VitB12) in samples from metastatic (M) and non-metastatic PanNENs (NM). A–C Comparison of the renal function indicators of patients, including estimated glomerular filtration rate (eGFR, A), blood creatinine (Cr, B) and blood urea nitrogen (BUN, C). D Comparison of serum VitB12 concentrations in samples from metastatic (M) and non-metastatic PanNEN (NM).Supplementary file2 (TIF 7401 KB) [file 18_2023_5084_MOESM2_ESM.tif]

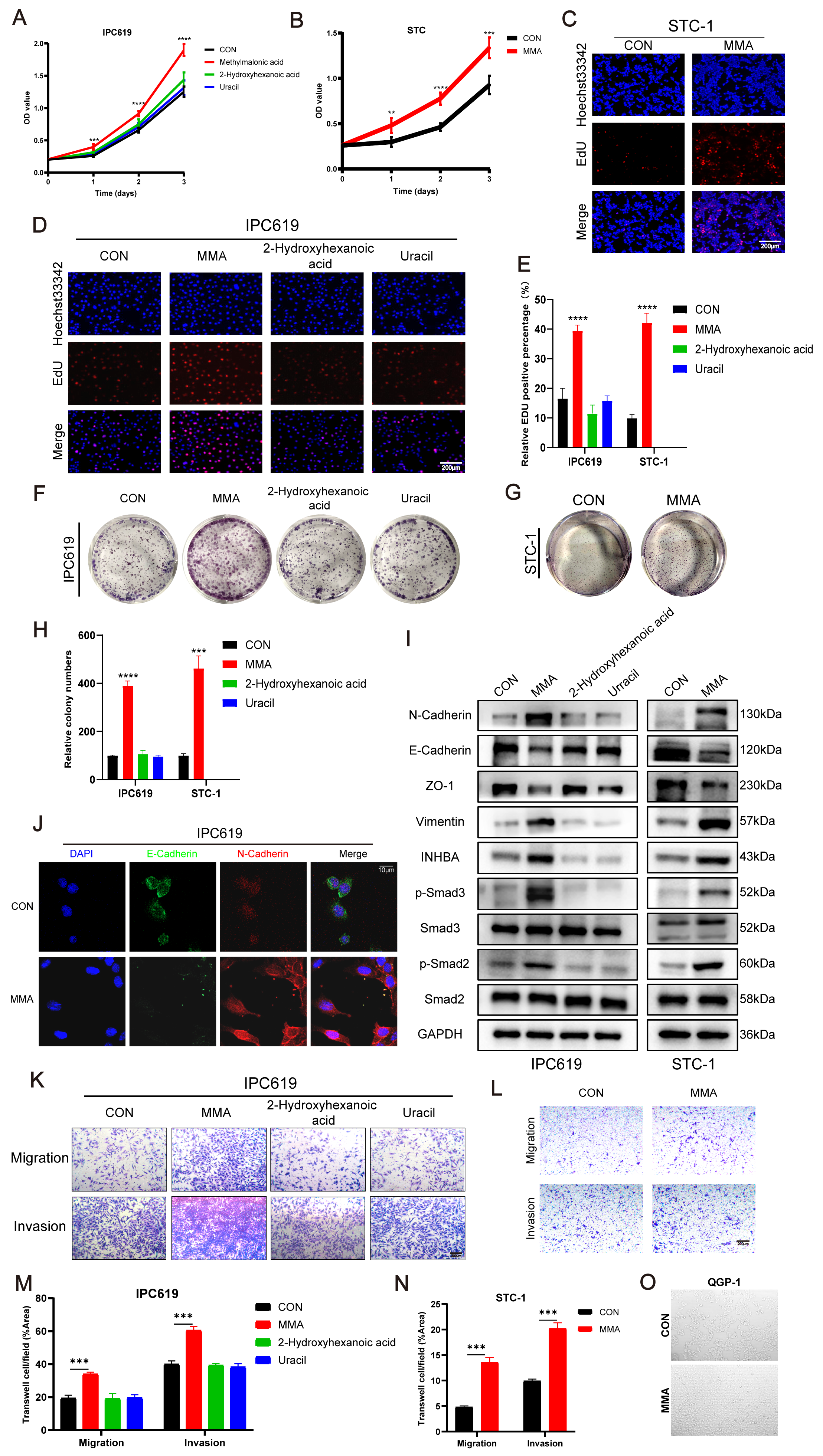

Supplement: Supplementary file 3 — MMA promotes proliferation and EMT mediated migration and invasion of neuroendocrine tumor cells. A–H The effects of the three significantly elevated metabolites on cell proliferation were tested by cell counting CCK-8 (A, B), EDU assays (C–E), and colony formation (F–H) in IPC619 and STC-1 cells. I Western blots showed that only MMA induced EMT and increased the expression of INHBA, p-Smad2 and p-Smad3 among the three upregulated metabolites in IPC619 and STC-1 cells. J Typical IF images of the expression of E-cadherin and N-cadherin for IPC619 cells. K–N Transwell assays indicated that only MMA significantly increased cell migration and invasion in both IPC619 (K) and STC-1 (L) cells compared with the control groups. Statistics of migration and invasion cells in the transwell assays after treatment for 48 h were analysed (M, N). O Typical brightfield images to show changes in the morphology for QGP-1 cells induced by MMA compared with control.Supplementary file3 (TIF 32164 KB) [file 18_2023_5084_MOESM3_ESM.tif]

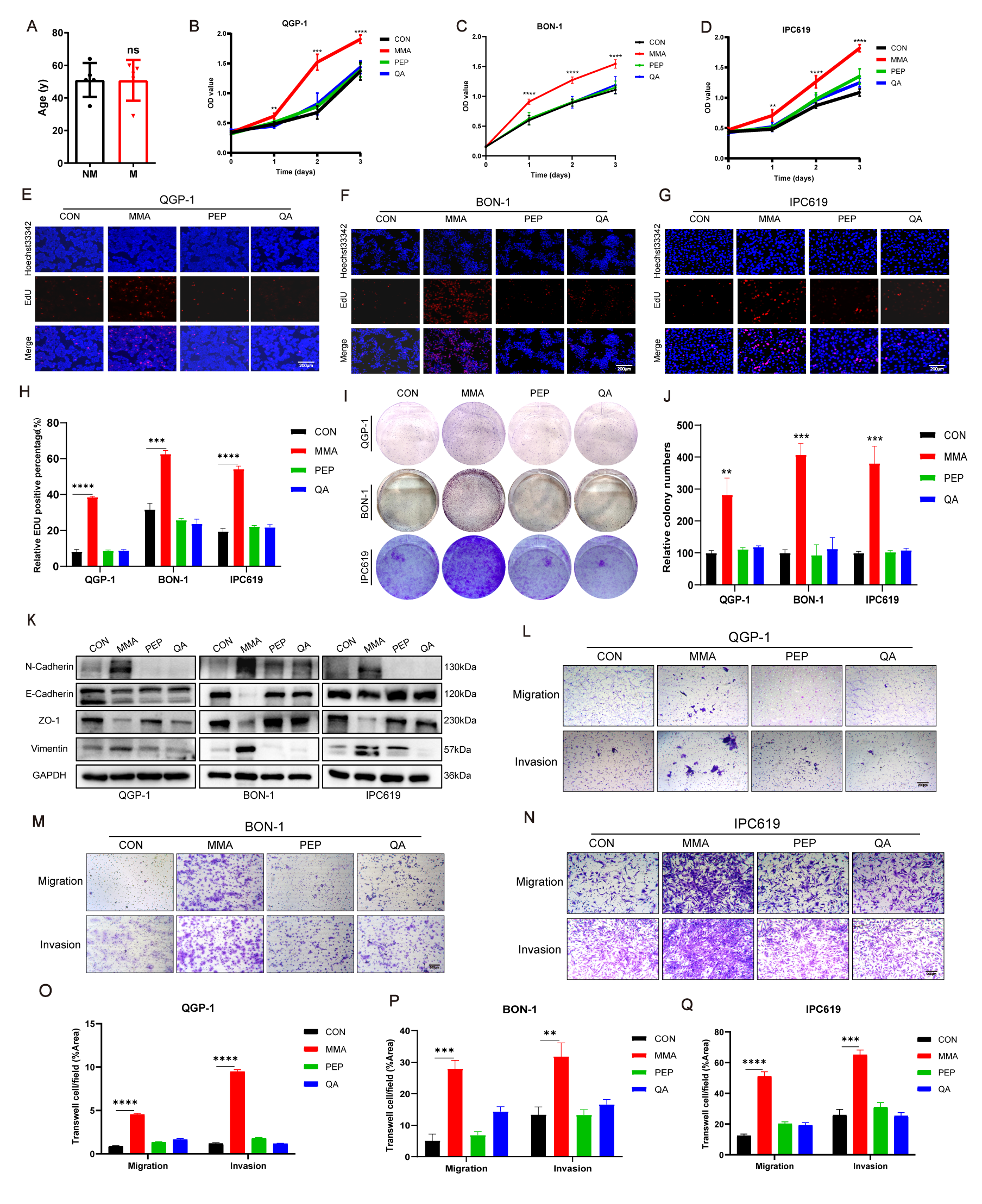

Supplement: Supplementary file 4 — Analysis of PanNEN cells treated with the three upregulated metabolites of aged serum. A There was no significant difference in ages of metastatic and non-metastatic PanNEN patients. B–J The effects of the three significantly upregulated metabolites of aged serum on cell proliferation were tested by cell counting CCK-8 (B–D), EDU assays (E–H) and colony formation (I, J) in QGP-1, BON-1 and IPC619 cells. K Immunoblots of EMT markers in QGP-1, BON-1 and IPC619 cells treated with the three metabolites. L–Q Transwell assays indicated that only MMA significantly increased cell migration and invasion in QGP-1 (L), BON-1 (M) and IPC619 (N) cells instead of phosphoenolpyruvate (PEP) or quinolinate (QA). Statistics of migration and invasion cells in the transwell assays after treatment for 48 h were analysed (O–Q). Supplementary file4 (TIF 14996 KB) [file 18_2023_5084_MOESM4_ESM.tif]

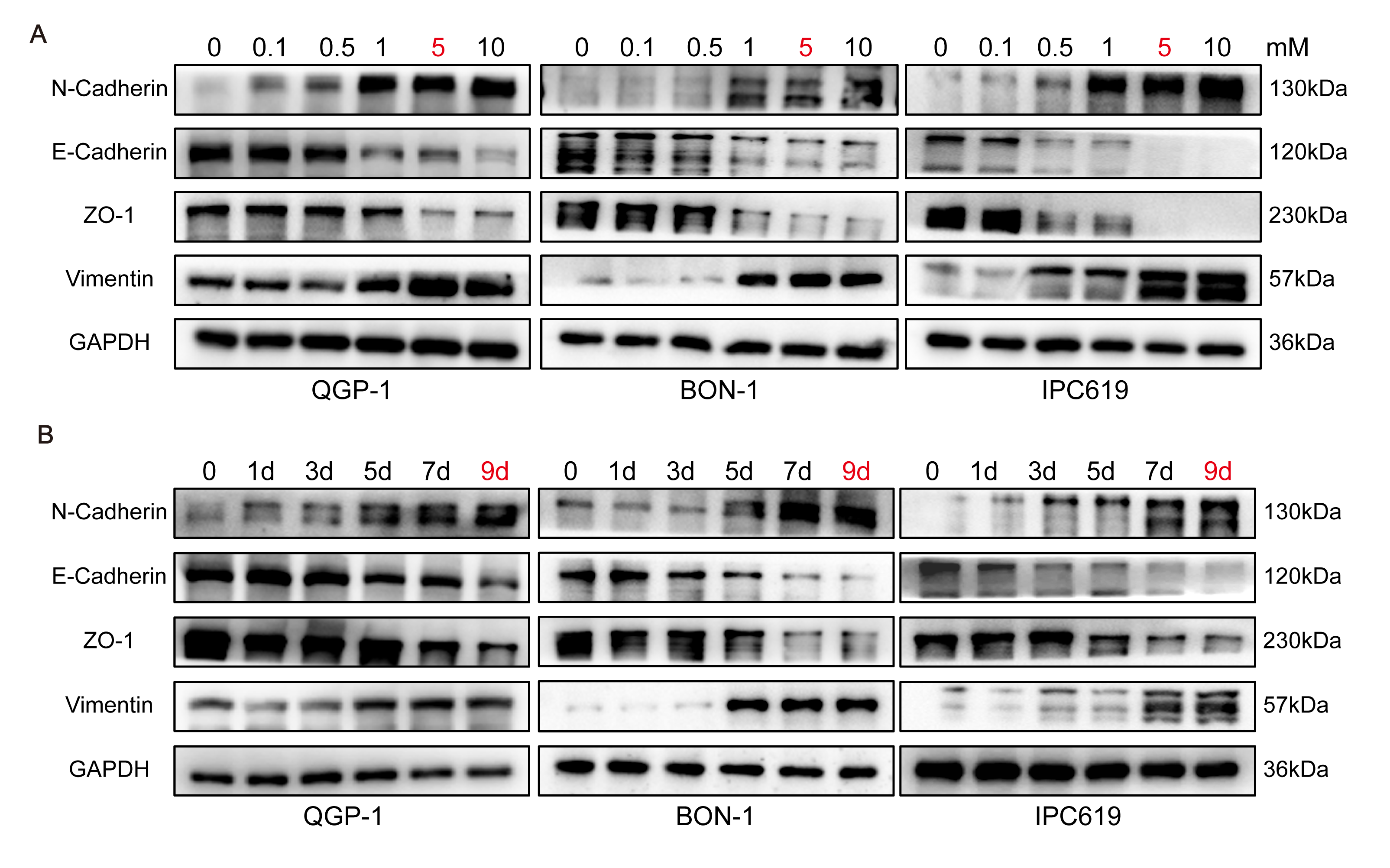

Supplement: Supplementary file 5 — Determination of optimal conditions for MMA stimulation of PanNEN cells. A Immunoblots of EMT markers in QGP-1, BON-1 and IPC619 cells treated with indicated concentrations of MMA. B Immunoblots of EMT markers in QGP-1, BON-1 and IPC619 cells treated with 5mM MMA for indicated time. Supplementary file5 (TIF 25364 KB) [file 18_2023_5084_MOESM5_ESM.tif]

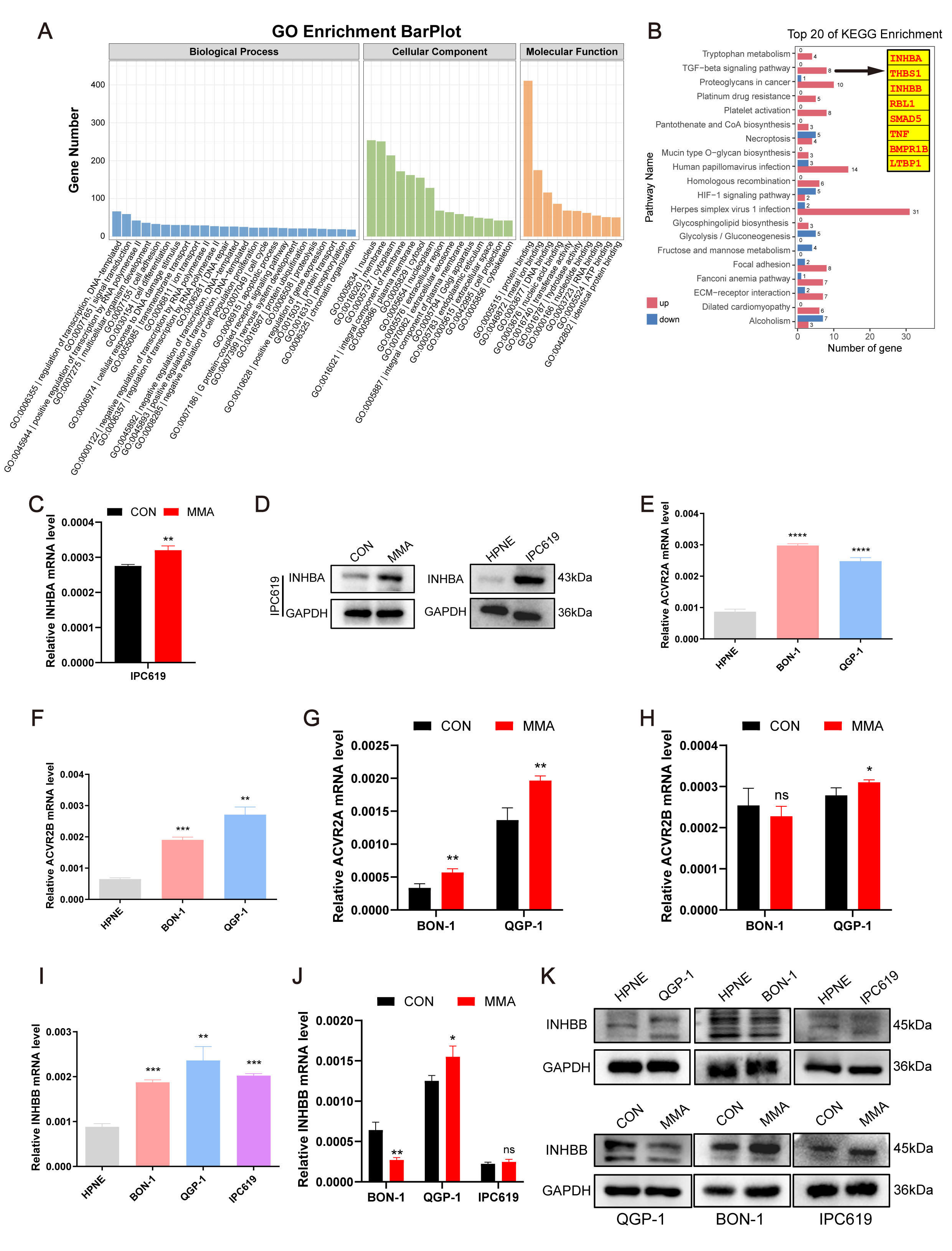

Supplement: Supplementary file 6 — RNA seq analysis on MMA-induced QGP-1 cells. A GO analysis showed that MMA induced a series of biological processes in QGP-1 cells. B There were 8 up-regulated genes enriched in TGF-β pathway in MMA-induced QGP-1 cells. C QPCR was performed to detect the relative expression of INHBA in MMA treated and the control group in IPC619 cells. D The expression of INHBA in indicated groups were evaluated by western blotting. E, F The relative expression of ACVR2A (E) and ACVR2B (F) in human normal HPNE and PanNEN cells (QGP-1 and BON-1) was detected by qPCR assay. G, H The relative expression of ACVR2A (G) and ACVR2B (H) in MMA treated and the control group in QGP-1 and BON-1 cells. I The relative expression of INHBB in human normal HPNE and PanNEN cells (QGP-1, BON-1 and IPC619) was detected by qPCR assay. J QPCR was performed to detect the relative expression of INHBB in MMA treated and the control group in QGP-1, BON-1 and IPC619 cells. K Immunoblots of INHBB expression in indicated groups. Supplementary file6 (TIF 27152 KB) [file 18_2023_5084_MOESM6_ESM.tif]

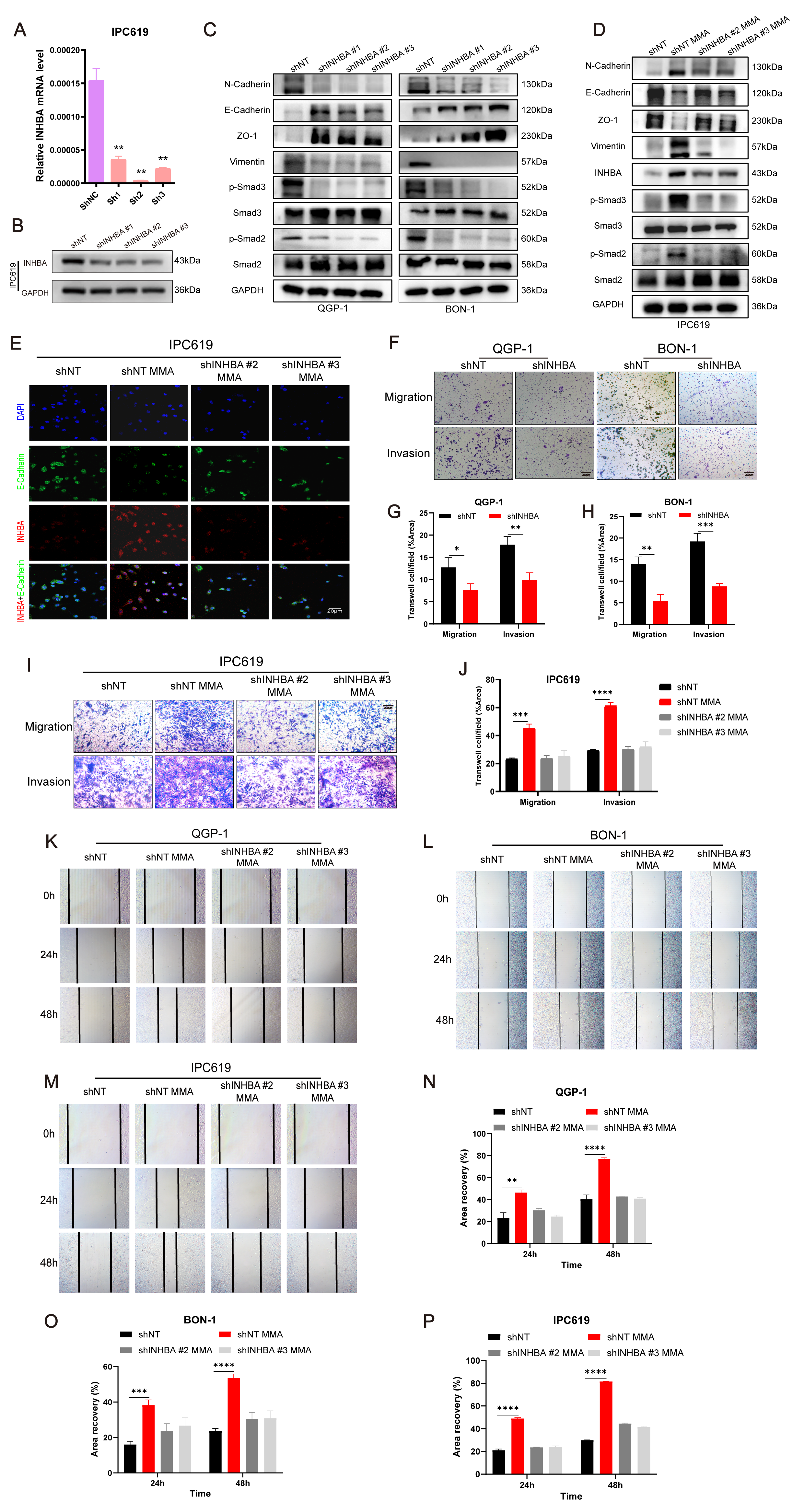

Supplement: Supplementary file 7 — INHBA is required for MMA to promote PanNEN cell progression. A INHBA levels in IPC619 cells transfected with shRNA lentivirus against INHBA or negative control were evaluated by qPCR. B Immunoblots of INHBA expression in IPC619 cells transfected with shRNA lentivirus against INHBA or negative control. C Immunoblots of EMT-related markers, p-Smad2 and p-Smad3 in QGP-1 and BON-1 cells transfected with shRNA lentivirus against INHBA or negative control. D Immunoblots of IPC619 cells with INHBA knockdown and treated with 5 mM MMA for 10 days. E Typical IF images of the expression of E-cadherin and INHBA for IPC619 cells. F–J Transwell migration/invasion assays of PanNEN cells. INHBA knockdown decreased the migrated and invasive ability of QGP-1 and BON-1 cells (F–H). INHBA was required for promotion of cell migration and invasion in MMA-induced IPC619 cells (I, J). K–P Wound healing assays showed that MMA induced cell migration was attenuated by the knockdown of INHBA in QGP-1, BON-1 and IPC619 cells. Supplementary file7 (TIF 43631 KB) [file 18_2023_5084_MOESM7_ESM.tif]

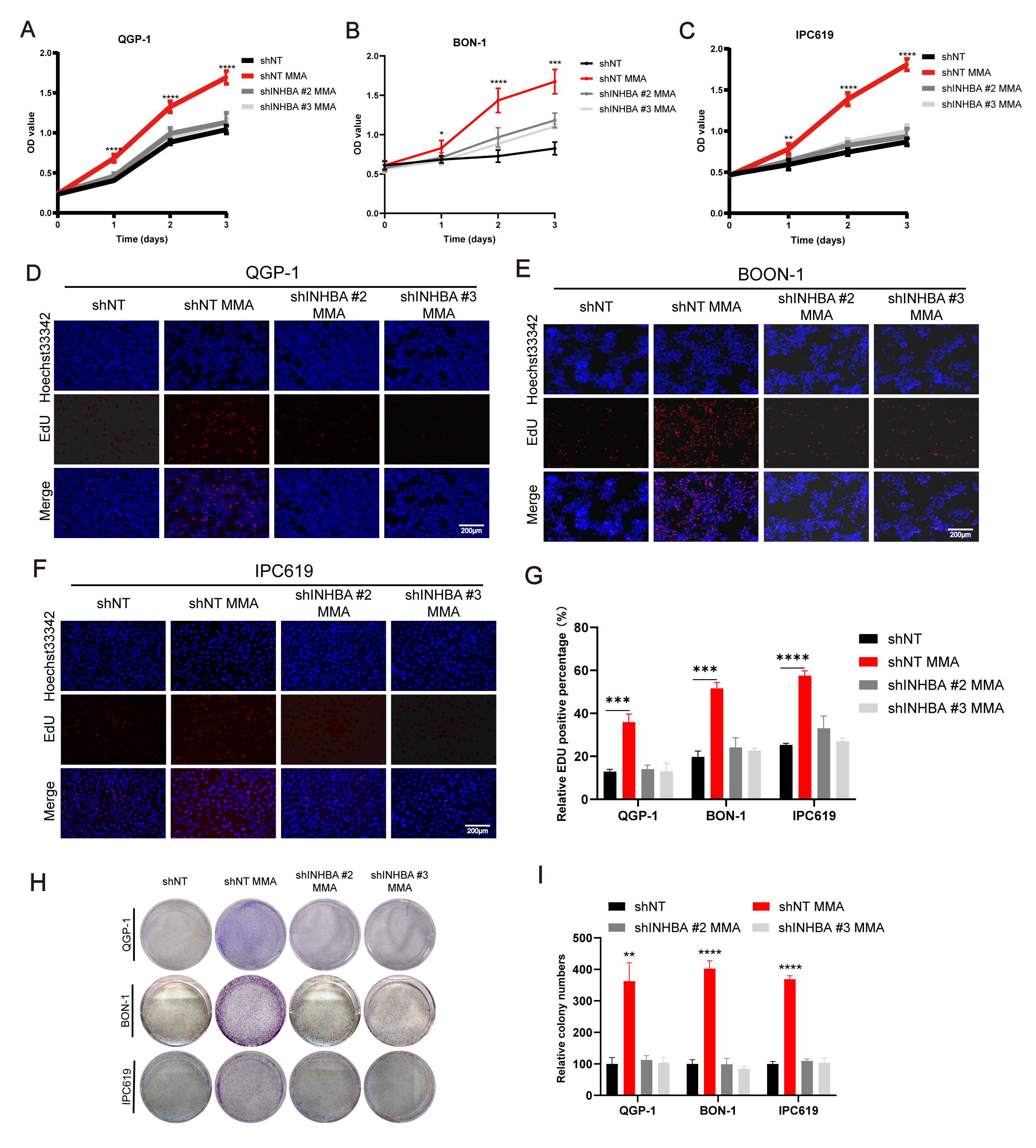

Supplement: Supplementary file 8 — INHBA knockdown decreases the MMA induced proliferation in PanNEN cells. Knockdown of INHBA with shRNA attenuated the role of MMA on proliferation detected by cell counting CCK-8 (A–C), EDU assays (D–G) and colony formation (H, I) in QGP-1, BON-1 and IPC619. Supplementary file8 (TIF 23863 KB) [file 18_2023_5084_MOESM8_ESM.tif]

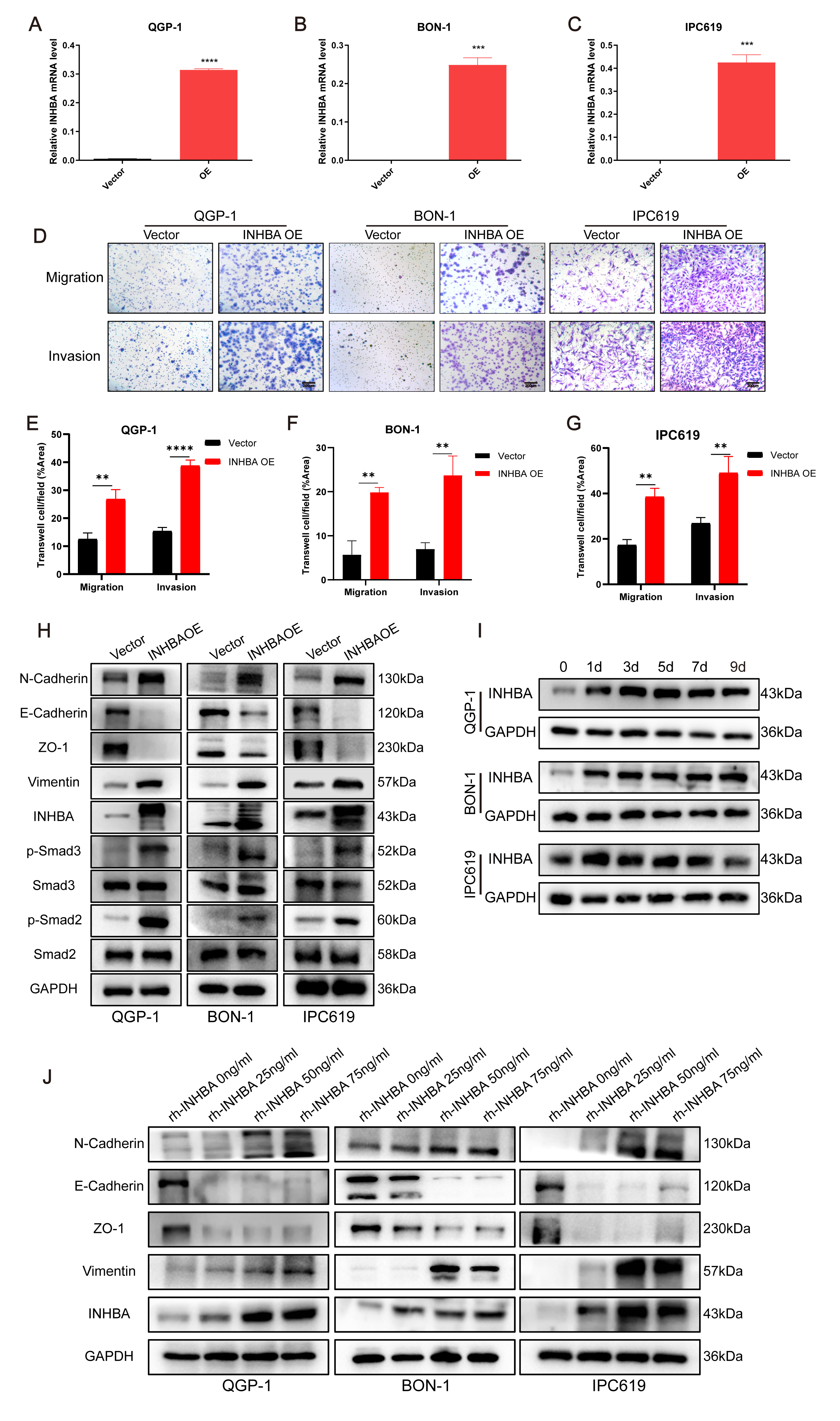

Supplement: Supplementary file 9 — Increased INHBA expression promotes PanNEN cell progression. A–C QPCR was performed to detect the efficiency of INHBA overexpression in QGP-1 (A), BON-1 (B) and IPC619 cells (C). D Overexpression of INHBA significantly increased cell migration and invasion in QGP-1, BON-1 and IPC619 cells when compared with the control groups. E–G Statistics of migration and invasion cells of the transwell assay after treatment for 48 h in QGP-1 (E), BON-1 (F) and IPC619 cells (G). H Western blots indicated that INHBA overexpression significantly increased the EMT markers, p-Smad2 and p-Smad3 in QGP-1, BON-1 and IPC619 cells. I Immunoblots of INHBA expression in QGP-1, BON-1 and IPC619 cells treated with MMA for indicated time. J Immunoblots of INHBA and EMT markers expression in QGP-1, BON-1 and IPC619 cells treated with recombinant human INHBA with indicated concentrations. Supplementary file9 (TIF 38785 KB) [file 18_2023_5084_MOESM9_ESM.tif]

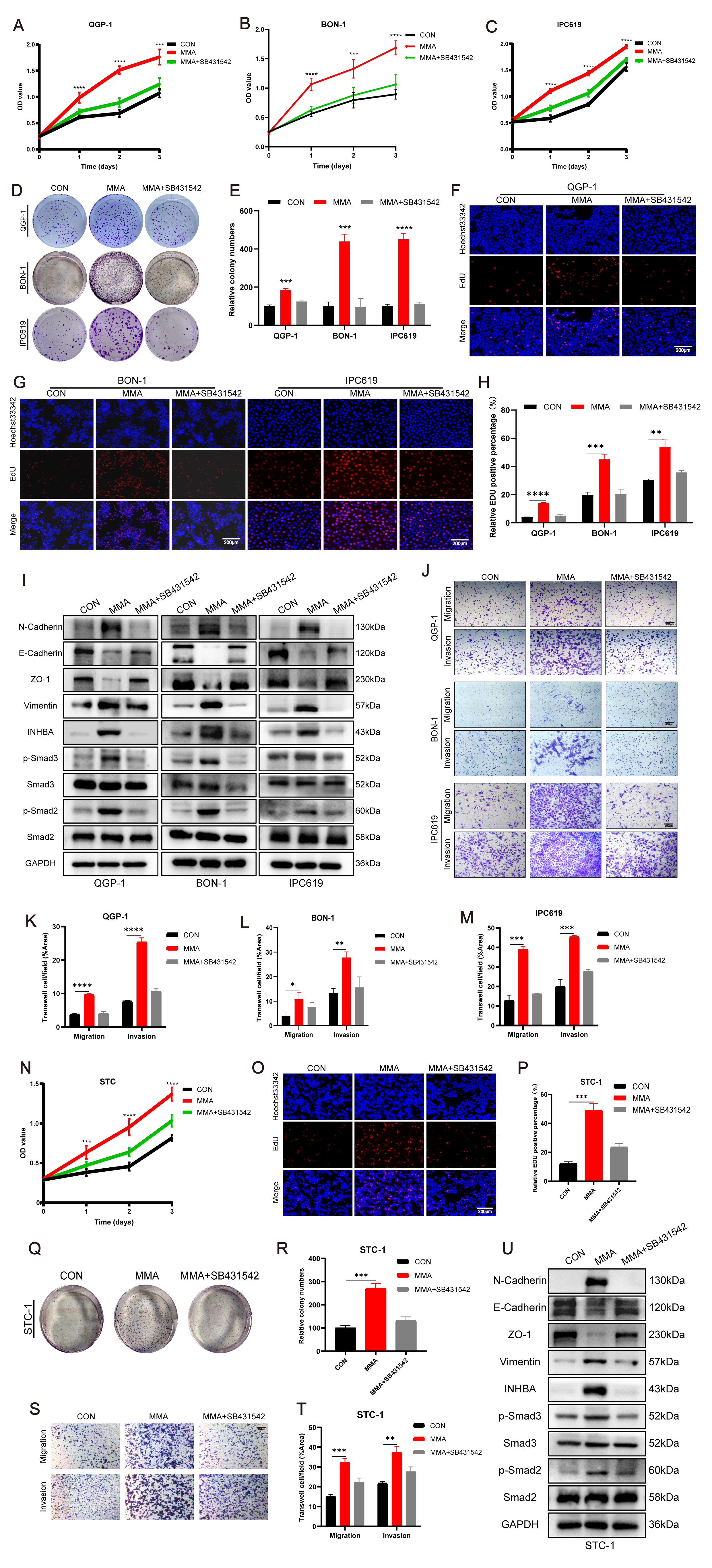

Supplement: Supplementary file 10 — Inhibitor SB431542 attenuates the MMA induced proliferation, migration and invasion of NEN cells. A–H Activin A signaling antagonist SB431542 decreased the role of MMA on proliferation detected by cell counting CCK-8 (A–C), colony formation (D,E), and EDU assays (F–H) in QGP-1, BON-1 and IPC619 cells. I Immunoblots of QGP-1, BON-1 and IPC619 cells with SB431542 and treated with 5 mM MMA for 10 days. J–M Transwell assays indicated that SB431542 attenuated MMA induced cell migration and invasion in QGP-1, BON-1 and IPC619 cells (J). Statistics of migration and invasion cells in the transwell assays after treatment for 48 h were analysed (K–M). N–R Inhibitor SB431542 decreased the role of MMA on proliferation detected by cell counting CCK-8 (N), EDU assays (O, P) and colony formation (Q, R) in STC-1 cells. S, T Transwell assays indicated that SB431542 attenuated MMA induced cell migration and invasion in STC-1 cells (S). Statistics of migration and invasion cells in the transwell assays after treatment for 48 h were analysed (T). U Immunoblots of STC-1 cells with SB431542 and treated with 5 mM MMA for 10 days. Supplementary file10 (TIF 49835 KB) [file 18_2023_5084_MOESM10_ESM.tif]

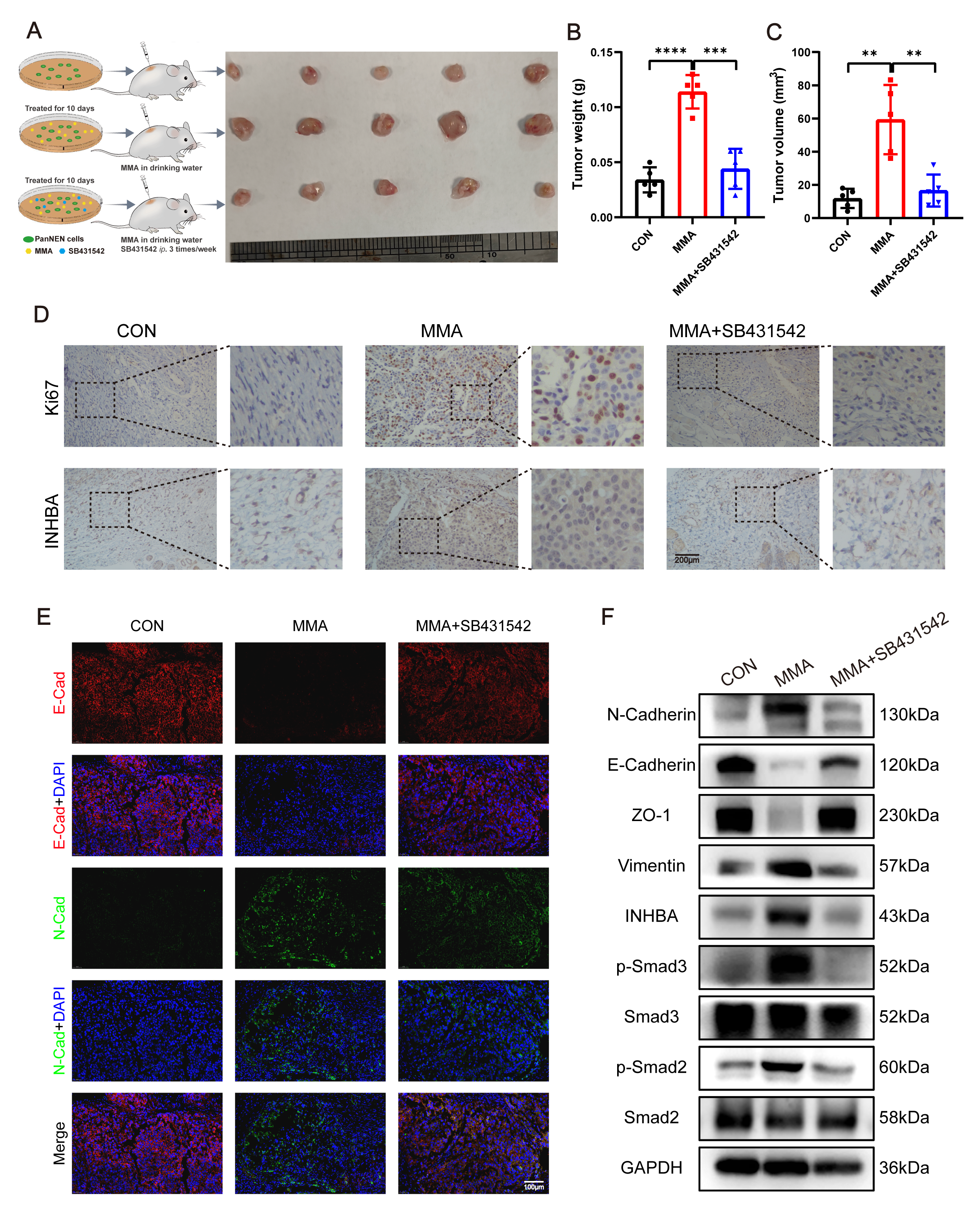

Supplement: Supplementary file 11 — Inhibitor SB431542 attenuates tumorigenesis induced by MMA in mice. A Schematic diagram of the MMA regimen in mice with subcutaneous PanNENs and general view of PanNEN tumorigenesis in nude mice subcutaneously injected with QGP-1 cells treated with ddH2O, MMA and MMA stimulation combined with SB431542. n = 5 mice per group. B–C Weight (B) and volume (C) of subcutaneous tumor of nude mice in three groups. D Typical images of IHC staining with Ki67 and INHBA in subcutaneous tumor of nude mice in three groups. E Typical IF images of the expression of E-cadherin and N-cadherin in subcutaneous tumor in three groups. F Immunoblots of EMT markers, INHBA, p-Smad2 and p-Smad3 expression in subcutaneous tumor of three groups. Supplementary file11 (TIF 32472 KB) [file 18_2023_5084_MOESM11_ESM.tif]

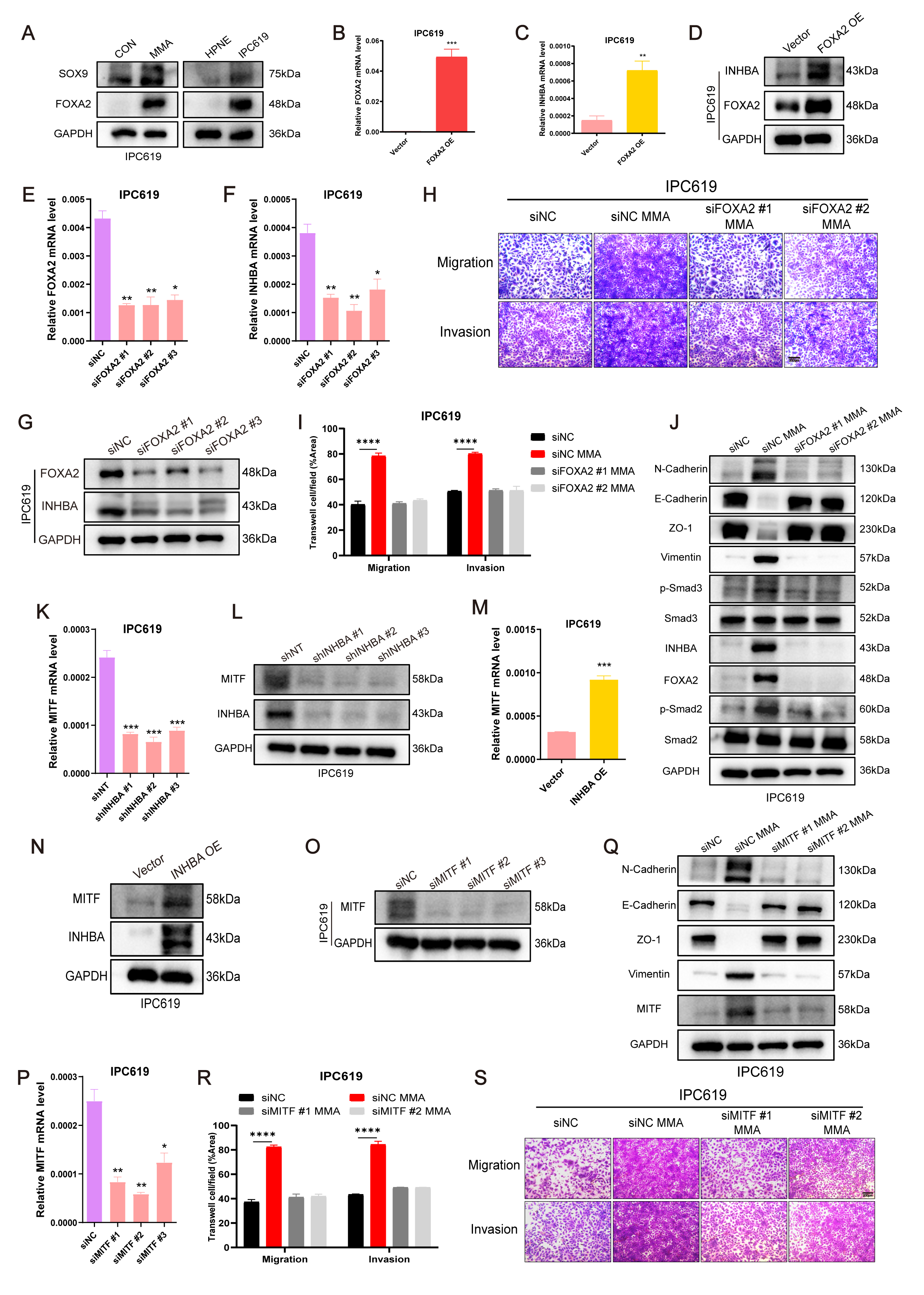

Supplement: Supplementary file 12 — MMA induces EMT in PanNEN cells by activating the FOXA2-INHBA-MITF axis. A Immunoblots of SOX9 and FOXA2 expression in indicated groups. B, C QPCR was performed to detect the efficiency of FOXA2 overexpression and the expression of INHBA in FOXA2 overexpressing groups compared with the control group in IPC619 cells. D Western blots indicated that FOXA2 overexpression significantly increased the expression of INHBA in IPC619 cells. E, F FOXA2 and INHBA levels in IPC619 cells transfected with FOXA2 siRNA or Scramble siRNA were evaluated by qPCR. G Immunoblots of FOXA2 and INHBA expression in IPC619 cells transfected with FOXA2 siRNA or Scramble siRNA. H, I Transwell migration/invasion assays of IPC619 cells. J Immunoblots of IPC619 cells with FOXA2 knockdown and treated with 5 mM MMA for 10 days. K MITF levels in IPC619 cells transfected with INHBA shRNA or Scramble shRNA were evaluated by qPCR. L Immunoblots of INHBA and MITF expression in IPC619 cells transfected with INHBA shRNA or Scramble shRNA. M MITF expression was evaluated in IPC619 cells transfected with INHBA overexpression or vector lentivirus by qPCR. N Western blots indicated that INHBA overexpression significantly increased the expression of MITF in IPC619 cells. O Immunoblots of MITF expression in IPC619 cells transfected with MITF siRNA or Scramble siRNA. P MITF levels in IPC619 cells transfected with MITF siRNA or Scramble siRNA were evaluated by qPCR. Q Immunoblots of IPC619 cells with MITF knockdown and treated with 5 mM MMA for 10 days. R, S Transwell migration/invasion assays of IPC619 cells. Supplementary file12 (TIF 31166 KB) [file 18_2023_5084_MOESM12_ESM.tif]
