# Supplementary material for: FOXA2-initiated transcriptional activation of INHBA induced by methylmalonic acid promotes pancreatic neuroendocrine neoplasm progression
Source: Cell Mol Life Sci. 2024 Jan 22;81(1):50. doi: 10.1007/s00018-023-05084-0 (PMC10803496; doi:10.1007/s00018-023-05084-0)
Supplement: Supplementary file 13 — Supplementary file13 (DOCX 18 KB) [file 18_2023_5084_MOESM13_ESM.docx]

**Supplementary materials**

**Supplementary Table1 Serum metabolomics analysis in patients with pancreatic neuroendocrine tumors**

**Supplementary Table2** **Primer sequence**

| **Gene** | **Primer sequence** |
| --- | --- |
| INHBA for qRT-PCR | F: CCTCCCAAAGGATGTACCCAA |
|  | R: CTCTATCTCCACATACCCGTTCT |
| INHBB for qRT-PCR | F: GTGAAGCGGCACATCTTGAG |
|  | R: GCGAAGCTGATGATTTCGGAAAC |
| ALK4 for qRT-PCR | F: CAGGATCGACTTGAGGGTGC |
|  | R: CGGCGATGATGCCTACCAG |
| ACVR2A for qRT-PCR | F: GTTTGCCGTCTTTCTTATCTCCT |
|  | R: GTCACCATAACACGGTTCAACA |
| ACVR2B for qRT-PCR | F: AGACACGGGAGTGCATCTACT |
|  | R: GCCTATCGTAGCAGTTGAAGTC |
| TGFB2 for qRT-PCR | F: CAGCACACTCGATATGGACCA |
|  | R: CCTCGGGCTCAGGATAGTCT |
| TGFBR2 for qRT-PCR | F: GTAGCTCTGATGAGTGCAATGAC |
|  | R: CAGATATGGCAACTCCCAGTG |
| FOXA2 for qRT-PCR | F: GGAGCAGCTACTATGCAGAGC |
|  | R: CGTGTTCATGCCGTTCATCC |
| SOX9 for qRT-PCR | F: AGCGAACGCACATCAAGAC |
|  | R: CTGTAGGCGATCTGTTGGGG |
| FOXD1 for qRT-PCR | F: TGAGCACTGAGATGTCCGATG |
|  | R: CACCACGTCGATGTCTGTTTC |
| FOXF2 for qRT-PCR | F: AATGCCACTCGCCCTACAC |
|  | R: CGTTCTGGTGCAAGTAGCTCT |
| FOXO1 for qRT-PCR | F: TCGTCATAATCTGTCCCTACACA |
|  | R: CGGCTTCGGCTCTTAGCAAA |
| NKX2-5 for qRT-PCR | F: CCAAGGACCCTAGAGCCGAA |
|  | R: ATAGGCGGGGTAGGCGTTAT |
| SOX5 for qRT-PCR | F: CAGCCAGAGTTAGCACAATAGG |
|  | R: CTGTTGTTCCCGTCGGAGTT |
| TOP2B for qRT-PCR | F: AGCCATTGACGCAGTTCATGT |
|  | R: CCTGGCACAAAGGTAACCTCC |
| FOXM1 for qRT-PCR | F: CGTCGGCCACTGATTCTCAAA |
|  | R: GGCAGGGGATCTCTTAGGTTC |
| MITF for qRT-PCR | F: CAGTCCGAATCGGGGATCG |
|  | R: TGCTCTTCAGCGGTTGACTTT |
| GAPDH for qRT-PCR | F: GGAGCGAGATCCCTCCAAAAT |
|  | R: GGCTGTTGTCATACTTCTCATGG |
| ACTB for qRT-PCR | F: CATGTACGTTGCTATCCAGGC |
|  | R: CTCCTTAATGTCACGCACGAT |
| INHBA for ChIP-qPCR | F: TGGATGAAAGCTGAAGGACA |
|  | R: GGTGTTTACCTGTGGCAGCA |

**Supplementary Table3 Antibody information (on next page)**

**Supplementary Table4 Gene differential expression of MMA induced and control QGP-1 cell**

**Supplementary Table5 Transcription factor prediction of INHBA**

**Supplementary Table6 Gene differential expression of INHBA overexpressed and control QGP-1 cells**

**Supplementary Table3 Antibody information**

| **Antibody** | **Company** | **Catalogue** | **Dilution ratio** |
| --- | --- | --- | --- |
| N-cadherin | Proteintech | 22018-1-AP | 1：1000 |
| E-cadherin | Proteintech | 60335-1-Ig | 1：1000 |
| ZO-1 | Proteintech | 66452-1-Ig | 1：1000 |
| Vimentin | CST | 5741T | 1：1000 |
| INHBA | Abcam | ab128958 | 1：1000 |
| p-Smad3 (Ser423/425) | CST | 9520T | 1：1000 |
| Smad3 | CST | 9523T | 1：1000 |
| p-Smad2 (Ser465/467) | CST | 18338T | 1：1000 |
| Smad2 | Proteintech | 12570-1-AP | 1：1000 |
| TGF-β2 | Proteintech | 19999-1-AP | 1：1000 |
| GAPDH | Proteintech | 60004-1-Ig | 1：5000 |
| INHBB | ORIGENE | TA350699S | 1：1000 |
| FOXA2 | Abcam | ab256493 | 1：1000 |
| SOX9 | Abcam | ab185230 | 1：1000 |
| MITF | Abcam | ab140606 | 1：1000 |
| NCAM1 | Abcam | ab220360 | 1：1000 |
| Chromogranin A | Proteintech | 10529-1-AP | 1：1000 |
| Synaptophysin | Proteintech | 17785-1-AP | 1：1000 |
| SSTR2 | Abcam | ab134152 | 1：1000 |
| SSTR5 | Proteintech | 66772-1-Ig | 1：1000 |
| ACTIN | Proteintech | 81115-1-RR | 1：5000 |
